# Metal Oxide Nanoparticles as Biomedical Materials

**DOI:** 10.3390/biomimetics5020027

**Published:** 2020-06-08

**Authors:** Maria P. Nikolova, Murthy S. Chavali

**Affiliations:** 1 Department of Material Science and Technology, University of Ruse “A. Kanchev”, 8 Studentska Str., 7017 Ruse, Bulgaria; 2 Department of Chemistry (PG Studies), Shree Velagapudi Ramakrishna Memorial College, Nagaram Guntur District, Andhra Pradesh 522 268, India; ChavaliM@gmail.com; 3 Nano Technology Research Centre, MC Education, Training, Research and Consutancy, Tenali, Guntur District, Andhra Pradesh 522 201, India

**Keywords:** metal oxides, nanotoxicity, oxidative stress, ROS, magnetic nanoparticles

## Abstract

The development of new nanomaterials with high biomedical performance and low toxicity is essential to obtain more efficient therapy and precise diagnostic tools and devices. Recently, scientists often face issues of balancing between positive therapeutic effects of metal oxide nanoparticles and their toxic side effects. In this review, considering metal oxide nanoparticles as important technological and biomedical materials, the authors provide a comprehensive review of researches on metal oxide nanoparticles, their nanoscale physicochemical properties, defining specific applications in the various fields of nanomedicine. Authors discuss the recent development of metal oxide nanoparticles that were employed as biomedical materials in tissue therapy, immunotherapy, diagnosis, dentistry, regenerative medicine, wound healing and biosensing platforms. Besides, their antimicrobial, antifungal, antiviral properties along with biotoxicology were debated in detail. The significant breakthroughs in the field of nanobiomedicine have emerged in areas and numbers predicting tremendous application potential and enormous market value for metal oxide nanoparticles.

## 1. Introduction

**Figure 1:**
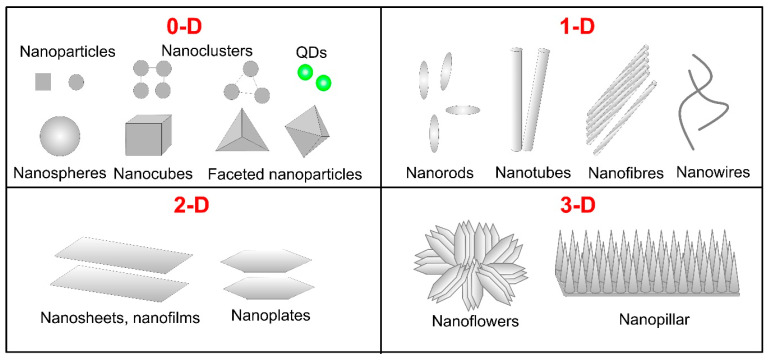
Scheme of zero-, one-, two- and three-dimensional nanostructured materials with different morphologies. 0-D, 1-D, 2-D, and 3-D indicate zero-, one-, two- and three-dimensional NPs, respectively.

Progress in nanotechnology and interdisciplinary research enables the production of nanosized materials with unique physical and chemical properties that make them suitable candidates for biomedical applications. Generally, nanotechnology includes synthesis and control of matter at dimensions of a few hundred nanometers that enable specific size-dependent properties [1]. Nanoparticles (NPs) dedicated to nanomedical applications ought to have a preferential size of less than 200 nm [2]. Due to their small size and large surface area, NPs show enhanced colloidal stability and, therefore, increased bioavailability demonstrating the ability to cross the blood-brain barrier, enter the pulmonary system and adsorb through endothelial cells [3]. Specifically, metal oxide NPs (MONPs) possess some advantages such as high stability, simple preparation processes, easy engineering to the desired size, shape and porosity, no swelling variations, easy incorporation into hydrophobic and hydrophilic systems and easy functionalization by various molecules due to the negative charge of the surface, that make them a promising tool for biomedical applications [4]. Since MONPs react with in vivo systems differently, depending on their size, shape, purity, stability and surface properties, it is necessary to characterize their morphology. Based on the number of dimensions, which are not confined to the nano-range, MONPs can be classified into zero-, one-, two- and three-dimensional [5] as represented in Figure 1. Zero-dimensional nanomaterials include NPs, nanoclusters, quantum dots and so forth that have all dimensions on the nanometer scale. For the one-dimensional NPs such as nanorods, nanotubes, nanowires, nanofibers, one dimension is outside the nanoscale. Two-dimensional or planar nanomaterials like nanosheets, nanocoatings, nanofilms, nanoplatelets, have two dimensions outside the nanoscale. Dendrimers, bundles of nanowires or nanotubes, nanopillars, nanoflowers, multi-nanolayers and so forth belong to the group of three-dimensional NPs. They are formed when nanomaterials aggregate in a certain size bigger than 100 nm in all three orthogonal directions. For the synthesis of these MONPs, various methods have been introduced that are summarized in previous reviews [6]. Generally, MONPs have highly ionic nature and can be organized with crystal morphologies exhibiting various reactive sites and corners. The deposition of MONPs on the desired position with a nanoscale resolution on a certain substrate has a promising potential to realize nanodevices for various applications in chemistry, electronics, optics and biomedicine [7].

The biomedical application of NPs for diagnostics and therapeutics (drug delivery and enhanced performance of medical devices) rapidly progresses during recent years. The usage of MONPs for diagnostics and therapy offers many advantages of modern medicine. The engineering of water-dispersible NPs allows these particles to be used in countless basic or applied biomedical researches. Right now, NPs are used in diagnostic for imaging of plentiful molecular markers, of genetic and autoimmune diseases, malignant tumors, photosensitizers in photodynamic therapy and target delivery of drugs [8]. Today NPs for biomedical purposes are adopted as diagnostic imaging materials such as viable fluorescent-labeled particles (semiconductor quantum dots), magnetic resonance imaging (iron nanooxides) and so forth. This work was aimed at exploring the complications to induce further investigations by summarizing new approaches, benefitting the features of MONPs and successful studies of applications of nano metal oxides in nanomedicine. A special interest has been shown towards green synthesized nano-oxides and their antibacterial activity as well as towards the toxic effects of MONPs on the organism.

## 2. Physical-Chemical Properties of Nano-Oxides

When entered into the body, NPs interact with biofluids and cell biomolecules that facilitate the physical transfer of the particles into the inner cellular structures [9]. As the biological response to NPs depends on the variety of factors such as size, morphology, aggregation and so forth, the controlled synthesis techniques are focused on attaining of NPs with tailored morphological configurations, sizes, distribution and stability. The main characteristics influencing the performance of NPs when contacting with cell can be summarized as follow:

### 2.1. Shape and Size

The size determines the surface-to-volume ratio and can affect the biodistribution and material uptake [10]. Since the openings of the blood vessels in many tumors are less than 200 nm and the mammalian vasculature has a pore size of about 5 nm and below this size, Elsabahy and Wooley suggest that the intermediate size (20–100 nm) of NPs has the highest potential for in vivo application due to the ability to propose enough circulation time [11]. The hydrodynamic size is one of the most important factors in determining the distribution and clearance of NPs in the organism. Research reports suggested that iron oxide NPs >100 nm in diameter were rapidly trapped in the liver and spleen through macrophage phagocytosis whereas those with the size of <10 nm were eliminated through renal clearance [12]. Numerous experiments pointed out that smaller than 5 nm NPs overcome cell barriers via translocation or other nonspecific mechanisms while larger particles entered the cell by pinocytosis, phagocytosis or other specific or non-specific cell transport mechanisms [8] Regarding the shape, nanosized oxides with different morphologies such as nanorods, nanospheres, nanocubes, nanowires, nanotubes and so forth have been synthesized. In vitro evaluation on iron oxide nanorods and nanospheres carried out in human carcinoma cells indicated that hematite nanorods were more quickly and in a higher extend internalized than nanospheres [13]. The more favorable cellular uptake of rod-shaped NPs, as opposed to spherical NPs, Andrade et al. [14], explained with the larger area of contact between the cell membrane and rod-shaped NP. Comparing tripod with spherical iron oxide NPs, it has been found that tripod particles had lower cytotoxicity in HeLa and Hepa 1-6 cells at concentrations between 0.022 and 0.35 mg Fe/mL [15]. Other authors showed that the internalization of nanoflowers was higher than single-core NPs [16]. It follows that the effect of the shape on NP toxicity is relevant to high aspect ratio conditions. The different combinations of shapes and sizes with specific properties of nanomaterials have to be also considered.

### 2.2. Surface Area and Surface Energy

The increased surface area and energy of NPs result in a drastic reduction of thermodynamic stability hindering the degree of uniformity, shape and size [17]. The surface of crystal oxide NPs consists of oxygen atoms with a lower coordination number due to the disruption of the crystal periodicity that leads to violation of the electro-neutrality between anions and cations and, therefore, surface change [18]. As NPs become smaller the percentage of surface located atoms increases concerning the total amount of atoms. The numerous edges and corners of the particles are potential reactive surface sites. The high surface-to-volume ratio provides NPs with a large number of active sites, small size and particular shape that could control reactivity. The particles with a larger surface area have a higher percentage of interaction as compared to larger particles when contacting the cell [19].

### 2.3. Crystal Structure

The most common cause of the toxic effects of NPs while interacting with cells is the release of metal ions [8]. The dissolution is influenced by crystallinity, crystal phase, surface strain, size, defects, media composition and so forth. Since NPs have a greater fraction of atoms at the corners and edges, this makes it easier for ions from the surface to break away from NPs due to high free energy [20]. For example, due to the variation in surface Gibbs free energy of different faces, the dissolution kinetics of polar (0001) terminated ZnO NPs in pure water was higher than of non-polar (1010) crystal surfaces [21]. In this context, He et al. [22] found that (0001) face had 3 times higher oxygen vacancy abundance in the bulk than in (1010) surfaces.

Nano TiO_2_ containing amorphous phase was more soluble than crystalline TiO_2_ while pure anatase NPs were more soluble than mixed anatase-rutile NPs [23]. Similarly, in an artificial media at neutral (pH 7) and low (pH 1.5) pH, nano-anatase displayed greater solubility compared to nano-rutile [24]. However, Gurr et al. [25] discovered that rutile type TiO_2_ NPs damaged DNA and triggered chromosome segregation and membrane changes, while anatase TiO_2_ NPs with the same sizes were found to be non-toxic. It follows that the complexity of toxicity assessment should account for other factors such as aggregation, shape, ROS formation and the complexity of bioenvironment and bio interactions, where organic or ionic molecules may form soluble or non-soluble complexes with the released ions that can control bioavailability.

### 2.4. Dispersibility and Aggregation

Because of the van-der-Waals forces, higher surface energy, and/or magnetic attraction, NPs show a sharp tendency toward agglomeration and when used at a concentration of 1000 ppm, MONPs tend to form aggregates [26]. The degree of aggregation influences biodistribution, biological and biomedical activities of nanooxides. For example, Lousinian et al. [27] discovered that the small aggregate size of ZnO NPs triggered high fibrinogen adsorption versus larger aggregate size due to the inverse relation between aggregate size and surface area. It was also concluded that the aggregations of very small MgО NPs (~5 nm) could reduce the efficiency of interaction with bacteria [28]. For that reason, the engineered NPs are usually stabilized in colloidal suspension by organic or inorganic compounds such as carbonate, cysteine or surfactants and they determine the surface charge of the whole system at different pH values. When MONPs have not stabilized appropriately, the particles may aggregate due to the colloid instability that could impede or slow their clearance and trigger eventual toxicity [29].

### 2.5. Surface Properties

The toxicity of the positively charged NPs is thought to be higher than that of negatively charged because of the electrostatic attraction with the negatively charged cell membrane. However, the positively charged NPs displayed an enhanced capacity for opsonization (adsorption of plasma proteins) [30] and their interaction with antibodies, serum proteins and so forth, might change the conformation of the adsorbed molecules leading to a change in their activity. There have been reported a correlation between the surface charge and the cellular uptake because positively charge NPs had higher mineralization rates in human breast cancer compared to negatively charged but both NPs were internalized equally into human umbilical vein endothelial cells [31]. Xiao et al. [32] showed that higher charged micellar NPs were massively incorporated by macrophage while NPs with a slight negative charge indicate high tumor uptake and low macrophage clearance. Additionally, the surface properties of MONPs are characterized by their zero-point of charge and acidity constant [18,19,20,21,22,23,24,25,26,27,28,29,30,31,32,33] values that were closely related to the particle aggregation.

### 2.6. Photocatalytic Activity

The most agreed mechanism of photocatalysis is related to the formation of holes trapped into surface defects and electrons localized into small hydrogen-rich areas. The charge separation minimizes the chance for hole-electron recombination and increases the photocatalytic, electrochemical activity and antibacterial effect [34]. The positive holes become trapped by water molecules from the air or solution that is oxidized producing •OH radicals which are powerful reactive oxygen species (ROS). The electrons in the conduction band can also trap oxygen which is reduced to form superoxide (O^2−•^) radicals that combine with H^+^ and produce peroxide radicals (•OOH) or hydrogen peroxide (H_2_O_2_) [35]. Some MONPs such as ZnO can generate superoxide, hydroxide radicals and singlet oxygen while others can produce one, two or no ROS molecules [36] or even inhibit ROS production induced by H_2_O_2_ which suggests anti-oxidant potent [37].

### 2.7. Chemical Composition

Nanoparticle-induced toxicity is found to depend on the MONPs type which could be attributed to NPs ability to generate ROS molecules and to release metal ions as mentioned above. For example, ZnO, SiO_2_, TiO_2_ and Al_2_O_3_ NPs of the same size (around 20 nm) demonstrated different toxicity on human fetal lung fibroblast (HFL1) [38]. After 48 hours of treatment at concentrations of 0.25, 0.50, 0.75, 1.00 and 1.50 mg mL^−1^, MTT analysis showed that ZnO NPs were more toxic to HFL1 followed by TiO_2_, SiO_2_ and Al_2_O_3_ in descending order. Other study demonstrated that copper and zinc oxide NPs appeared to be more toxic to two human pulmonary cell types while titania, alumina, ceria, and zirconia showed low to moderate toxicity without indicating a correlation between toxicity and either specific surface area or equivalent spherical diameter [39];

### 2.8. Target Cell Type

Different cell target specificity commonly displays different metabolic activity and therefore, different cell death mechanisms and sensitivity to MONPs exposure. When comparing the toxic effects of nano SiO_2_ on human monocytes (THP-1) and human lung epithelial cells (L-132), it was found that SiO_2_ NPs were more cytotoxic on THP-1 cell than on L-132 [40]. The cellular target specificities could be attributed to the function of phagocytosis which characterizes monocytes but not lung epithelial cells. Comparing the dose-dependent and time-dependent cytotoxic effects of different MONPs on two cell types – alveolar (A549) and distinguished monocytes to macrophages (THP-1) cells, A549 cells showed less sensitivity than THP-1 cell [39]. The higher sensitivity for macrophage responses and their superior capability to take part in particle aggregates via phagocytic mechanisms were expected to increase the macrophage responses to NPs.

## 3. Applications of MONPs in Biomedicine

To be used for a particular application, MONPs should meet certain requirements. For example, the MONPs implemented as drug carriers should have kinetics complying with the requirements for treating a certain infection and they have to be biodegradable to exclude further surgical intervention. Although a broad spectrum of MONPs is available, only TiO_2_, ZnO, CuO, ferric oxide (Fe_2_O_3_) and ferrous oxide (Fe_3_O_4_) appeared as comparatively safe for mammals [41].

### 3.1. Internal Tissue Therapy

**Figure 2:**
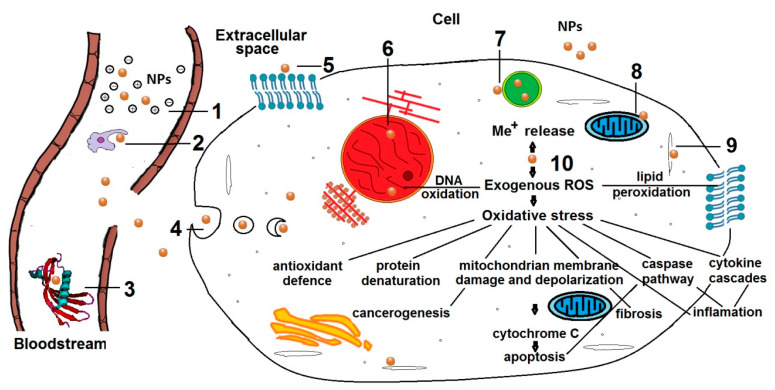
Mechanisms of metal oxide nanoparticles (MONPs) delivery pathway and cell damage in eukaryotic cells: (**1**) interaction with ions in circulation; (**2**) ingestion by phagocytic cells; (**3**) opsonization or enzymic degradation; (**4**) internalization via endocytosis after extravasation to the extracellular space or (**5**) membrane perforating and damage of its components and their function; (**6**) chromosomal aberrations and changes in cell replication rate; (**7**) lysosome rupture; (**8**) mitochondria damage; (**9**) lower growth rate, structural changes and shorten lifetimes of microtubules of the cytoskeleton; (**10**) generation of ROS, oxidative stress and subsequent processes.

The implementation of therapeutics is usually closely related to the capacity to affect diverse molecular signaling pathway that regulates the expression of growth factors, division, cell differentiation, migration and apoptosis [36]. MONPs could penetrate the body by respiration, ingestion, through the skin or by infusion or direct injection or transportation with nanofibers to a certain organ. The great advantage of nanomedicine for disease therapy constitutes the potential to create a nanocarrier structure with enhanced delivery efficiency due to the ability to translocate through the cell membrane [4]. The benefits of developing nanocarriers as drug-delivery systems include enhanced pharmacological activity, sustained and target delivery of more than one therapeutic agent, stability and bioavailability [42]. Engineering materials allow for improving the specificity of the nanosystems thus decreasing the side effects for patients. After entering the target cell, the toxicity of a certain nanoparticle does not give a regular pattern of changes. A summary of the circulation interactions and common mechanisms of MONPs induced cytotoxicity is shown in Figure 2.

The fate and cytotoxic effects of the MONPs may include:

- ingestion by the phagocytic cells (monocytes, macrophages and dendritic cells) that could pose a hazard to the cells [43,44];
- opsonization [45] or enzymic degradation;
- changes in cell membrane structure, integrity and disturbed function of its components influencing the cell transport [25,46];
- chromosomal segregations, aberrations, chromatin condensation and changes in the cell replication rate [38,47];
- hindered autophagy and macromolecules metabolization because of ruptured lysosomes which could activate apoptotic caspase pathways [48];
- disturbed production of energy through respiration and cellular metabolism by mitochondria damage [49];
- lower growth rate and cell division, structural changes and shorten lifetimes of microtubules of the cytoskeleton and hampered intracellular transport [50,51,52];
- generation of ROS and oxidative stress induces changes [47,53];
- production of ROS likely leads to protein denaturation, DNA oxidation and membrane lipid peroxidation which damages the cell integrity and influences the respiratory activity causing eventually cell death. Besides, in physiological conditions, ROS produced mainly by mitochondria mediate the intracellular signal transduction, regulate the protein phosphorylation and control intracellular Ca^2+^ homeostasis [54].

All the above-listed NPs’ triggered effects which damage the eukaryotic alter the cell adhesion, division, viability, differentiation, proliferation, migration, angiogenesis, and/or apoptosis. For that reason, the identification of the exact molecular mechanisms of NPs influence and control on the cell metabolism and functions is of utmost importance. The mechanisms of particles’ movement in the body right should also be specified in detail to open up the possibility of exact focusing of NPs towards target cells which will make their usage safer showing minimum side effects.

**Figure 3:**
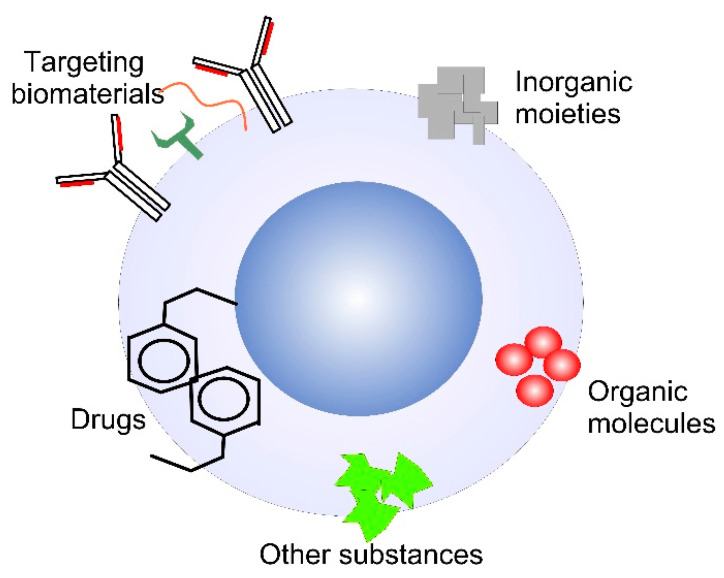
Scheme of possible modifications of MONPs for biomedical applications.

By targeting specific sites, NPs can reduce the overall dose of the medicament and thus to decrease the undesirable side effects. One of the challenges in targeting therapy with NPs is to reduce the undesired interaction with other molecules, toxic effects on normal tissue and to increase selectivity towards cancer cells or other target cells. This type of targeting therapy includes delivery and targeting nanocarriers linked with natural polymers such as polysaccharides, polyesters, DNA, RNA, polypeptides, enzymes, proteins and so forth or synthetic polymer materials conjugated with inorganic NPs – silica, MOs, HAP, and so forth (Figure 3). The coating shell is usually hydrophilic and makes the functionalized NPs compatible with bio-environment [55]. The surface coat determines the overall size of the colloid particle and is very important for the biokinetics and biodistribution of NPs in the body [56]. The shells could contain biological bioactive molecules such as organic acids, chitosan, gelatin or liposome coatings or polymeric materials like PEG, poly(vinyl alcohol) and so forth and surfactants (SDS, sodium oleate, etc.) that could protect, enhance or give additional effect and/or direct the NPs in the organism. The whole engineered system with a biological and non-biological origin that treats prevents or diagnosis a certain disease is called theranostics (diagnostic with target therapy). In that context, NPs should have a high loading capacity. The drugs could be covalently bonded to functionalize MONPs, for example, HAP [57] or to establish electrostatic interaction with the charged NPs. The medicaments linked to NPs could be anticancer, immunosuppressive, anticonvulsants, anti-inflammatory, antibiotics, antifungals, antiviral and alternative drugs. The ability of MONPs to localize the drug to the target cell may greatly enhance the use of these medicaments by reducing the necessary dose and sparing the normal tissue toxicity. The shell of the nanohybrid material could not only stabilize the MONPs but also eliminate the toxicity of NPs due to the formation of ROS, change their stay in the organism and make them tissue-specific [58]. The whole NPs should demonstrate good penetration and accumulation in the target cell and once the carriers are incorporated in the cell, they should be able to escape endosomes.

#### 3.1.1. Iron Oxide Nanoparticles

The magnetic properties of iron oxide NPs brand them suitable for magnetic separation of biological products and cells, diagnostics and guides for site-specific drug delivery [59]. Fe_3_O_4_ NPs are magnetic biomaterials that could be directed and concentrated by the external magnetic field and to be removed once the therapy is completed [60]. In the bloodstream, iron oxides NPs are usually subject to opsonization followed by recognition and elimination in the blood circulation [44]. The high vascularization and permeability of iron NPs trigger their uptake by the reticular endothelial system and make them recognizable by macrophages [43]. However, it is generally accepted that Fe_3_O_4_ NPs can kill cancer cells without compromising the regular cells. Since cancerous tissue is more sensitive to heat damage in vivo, magnetic NPs can target heat specifically to tumors by alternating magnetic fields, frictional or hysteresis heating [61]. This treatment is called hyperthermia. Hilger at al. [62] injected supermagnetic NPs into immunodeficient mice with implanted breast adenocarcinoma cells and after applying a magnetic field with 400 kHz frequency, the temperature within the tumor region rose to 73 °C. Based on the general presumption for penetration of NPs through various membranes, Hurbankova et al. [63] studied the impact of Fe_3_O_4_ NPs on the vascular system of the respiratory tract together with the inflammatory and cytotoxic parameters of bronchoalveolar lavage. The iron oxide NPs, compared to the control, induce an inflammatory response, cytotoxic damage and respiratory toxicity. The results further showed that Fe_3_O_4_ NPs after 28 days of installation were eliminated from the respiratory tract by the defense body mechanisms.

Iron is an important ion in all cells’ homeostasis. Due to the colloid instability of bare iron oxide NPs, different modifications improve the stability and prevent the opsonization (adsorption of plasma proteins) of NPs in blood circulation. The natural polymers such as the polysaccharide chitosan showing hypoallergenic, antibacterial and hemostatic properties, were used for the synthesis of chitosan-coated iron oxide NPs to develop drug delivery system to treat low bone mineral density like osteoporosis or slackening of the prosthesis [64]. The authors revealed that the majority of modified NPs were extracellularly located while the uncoated iron oxide NPs was predominantly found intercellular. The chitosan linked NPs amplified osteoblast proliferation, distinction and viability but at a concentration above 300 μg mL^−1^, the increased internalization of iron oxide NPs by osteoblasts induce apoptosis. Nevertheless, no concentration-dependent internalization of chitosan-coated NPs was witnessed suggesting fewer dosage restrictions in clinical practice. Other hemolytic and cell viability studies revealed that when iron oxide NPs were conjugated with BSA, improved specific absorption rate values were observed due to the enhanced colloidal stability and prevention of NPs aggregation [65]. Most hyaluronic acid (HA) drug conjugates with iron oxide NPs as a targeting moiety had been developed for cancer chemotherapy as macromolecular products [66].

Magnetic nanotubes allow not only magnetic properties but also differential functionalization of outer and inner surfaces which can be useful for magnetically assisted drug delivery. Yu et al [67] functionalized porous iron oxide nanorods with folic acid for target delivery of low water-soluble anticancer drug – doxorubicin (DOX) and found out that the presence of folic acid on the surface of nanostructures increase the cytotoxicity of doxorubicin and the cellular uptake by HeLa cells. This effect the authors attributed to the specific binding between folate receptors and folic acid which made these nanocarriers suitable for targeted drug delivery.

Similarly, various attempts had been made to control aggregation, anisotropy, specific absorption rate and so forth by doping MONPs not only with biomacromolecules such as HA, BSA, small interfering RNA (siRNA), proteins or chitosan but also with different synthetic polymeric coatings. Feng et al. [12] studied the in vivo and in vitro biological behavior of commercially available iron oxide NPs (10 and 30 nm) coated with PEG with positive surface charge and PEI which had an almost neutral charge. PEI coated NPs exhibited severe cytotoxicity contrary to macrophage and cancer cells while PEG-coated indicated no obvious cytotoxicity even at higher concentrations. Moreover, PEG-coated NPs were capable to induce autophagy which may have a defensive role against the cytotoxicity of iron oxide NPs. When drug-loaded, iron oxide NPs help to control the release of the medicament which could reduce the side effects due to their lower dosage and minimize or prevent the degradation of the drug because of the ability other pathways than gastrointestinal to be used [68].

**Figure 4:**
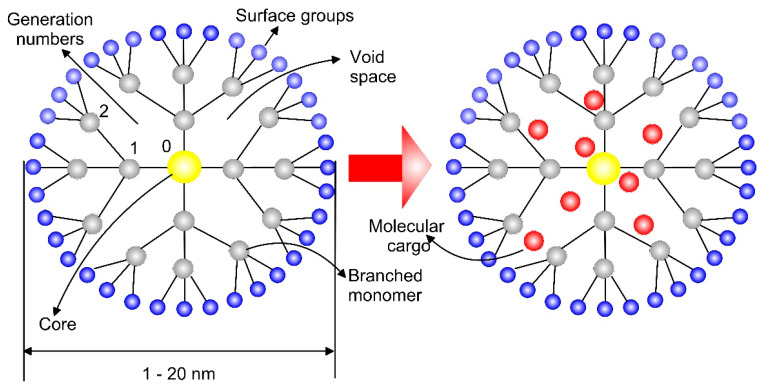
General representation of a model structure of a dendrimer and possible dendrimer-molecular cargo interactions.

Dendrimers are globular nanostructures with a core from MONPs or Au, encapsulating special molecules (drug, gene or cancer imaging) in their internal voids. A dendrimer can also perform solubility or dissolution enhancement and controlled delivery [69]. A schematic view of a typical dendrimer with its important parts, that are core, branches, generation numbers, functional groups and so forth, and dendrimer–cargo interactions are shown in Figure 4. Dendritic NPs such as PAMAM (poly(amidoamine)) are widely explored for drug delivery agents in cancer and antiviral therapy, gene delivery, medical imaging applications and vaccine delivery systems [70]. The quantity of the entrapped molecules depends on the size and shape of the dendrimer and the size of its internal cavities (Figure 4). These cavities are usually hydrophobic thus allowing for interaction with poorly soluble drugs or charged molecules like siRNA [71].

Polyvalent dendrimer-bearing magnetic NPs as carriers for siRNA delivery evaluated in a transgenic murine model of glioblastoma in vivo and in vitro promoted cytosolic release of endocytosed cargo more resourcefully than their components, resulting in an effectual delivery of siRNA to cell cytoplasm over a wide range of loading doses [72]. The non-covalent attachment of siRNA afforded dendriworms the ability to hold flexibility in siRNA loading without reformulation. The drug-release rate depends on the type of the chemical bond between the drug and carrier, the structure and steric effects of the conjugated NPs. The in vivo efficiency of anionic G4.5 PAMAM dendrimers with magnetic NPs as drug carriers for low water-soluble antipsychotic drug-risperidone was evaluated for the parameters of heart rate and brain development of Zebrafish larvae [73]. The most significant changes were observed when larvae were treated with free risperidone, not with the dendrimer NPs. This may indicate a decline in the side effects on the drug once administrated as a complex or decrease the effect on its onset. The toxicity of PAMAM Khodadust et al. attributed to the dense surface amine groups [74]. Consequently, suitable surface modification with drugs, vitamins, antibodies, PEG or imaging agents producing neutral or anionic surfaces can reduce the dendrimer biotoxicity. Other authors relate the mechanism of toxicity of the micellar NPs with their surface charge because the neutral surface charge has the longest circulation time and have lower uptake in the liver and spleen than positively and negatively charged NPs [75].

#### 3.1.2. Zinc Oxide Nanoparticles

Compared to normal cells, ZnO NPs showed selective cytotoxicity toward cancer cells in vitro and in vivo [76,77]. ZnO itself presents certain cytotoxicity in cancer cells due to higher ion release in acidic media and increased ROS induction and hence is used in cancer therapy. Evaluating the anticancer activity of ZnO NPs, Moghaddam et al. [49] observed apoptosis triggered by extrinsic and intrinsic apoptotic pathways in MCF-7 cancer cell line. The authors reported cell cycle arrest, a 5-fold increase in stress-responsive kinase inducing apoptosis and depolarization of the mitochondrial membrane potential indicating that ZnO NPs apoptosis was mitochondria-dependent. In human keratinocytes (HeCat) and epithelial cells (HeLa), commercial ZnO NPs and custom-built ZnO nanowires induced acute cytotoxic effects prompting actin filament bundling and structural changes in microtubules renovating them into rigid microtubules of tubulin [50]. ZnO NPs severely affect cell proliferation and survival because of acute cytoskeletal collapse triggering necroses followed by late ROS-dependent apoptotic processes.

As discussed previously, ZnO NPs are attractive candidates for cancer drug delivery because of their biodegradable characteristics [77]. Doxorubicin-ZnO nanocomplex was found to act as an efficient drug-delivery system against hepatocarcinoma cells enhancing the chemotherapy efficiency by increasing the intercellular concentration of doxorubicin [78]. Additionally, the nanocomposite showed excellent photodynamic therapeutic properties. Hollow ZnO spheres loaded with paclitaxel and functionalized with folic acid successfully induced cytotoxic effect against breast cancer cells in vitro and in vivo by reducing xenograft tumors in nude mice [79]. As in iron oxide NPs, to enhance the drug solubility, bioavailability and efficiency, Dhivya et al. [80] synthesized copolymer encapsulating ZnO NPs with hydrophobic PMMA-AA loaded with a large amount of hydrophobic drug curcumin. The results notified that the percentage release of curcumin was higher at pH 5.4 as opposed to pH 6 and pH 7.2. This fact is very important as the tumor cells are stable at about pH 6. Moreover, the Cur/PMMA-AA/ZnO NPs showed exceptional cytotoxicity contrary to ACS cancer cells compared to other bionanocomposite materials under similar experimental settings.

Since zinc participates in insulin synthesis, storage and secretion [81], ZnO NPs have also the potential to be effective anti-diabetic or alleviates diabetic complications agents when stabilized or conjugated with certain substances. In 2018, Hussein et al. [82] synthesized conjugated ZnO NPs (20–27 nm) with naturally biodegradable and biocompatible polymer-hydroxyethyl cellulose (HES) by a wet chemical process. With the purpose to attenuate the diabetic complications, male albino rats were treated with the conjugated ZnO NPs. The results indicated that CPR, pro-inflammatory IL-1α and ADMA levels increase meaningfully concomitant with a reduction in NO (signaling molecule for regulating angiogenesis, protein kinase G activity, protein phosphorylation and other processes connected with cell proliferation, migration and vasodilatation) level in the diabetic rats while ZnO NPs supplementation suggestively attenuated these factors which make ZnO particles very promising for enhanced selectivity towards atherosclerosis.

#### 3.1.3. Titanium Dioxide Nanoparticles

TiO_2_ is a prevalent material for biomedical applications used mainly in bone and tissue engineering owing to its ability to induce cell adhesion, osseointegration [83], cell migration and wound healing [84]. In vivo experiments with non-modified TiO_2_ upon 365 nm light irradiation indicated tumor growth suppression in glioma-bearing mice together with higher mice survivability [85]. Nitrogen-doped anatase NPs revealed higher visible light absorbance than neat TiO_2_ and at a concentration of 0.5 mg mL^−1^ resulted in 93% cell death of melanoma cells under UV light [86]. The authors also found that depending on the type of cancer, the sensitivity of cancer cells to modified NPs may vary significantly. However, the penetration of UV light through tissues is low and harmful and, therefore, photodynamic therapy related to tissue overhealing is significantly limited [87].

When titania nanotube arrays with 100 ± 10 nm diameter were decorated with TiO_2_ NPs, a significant increase in the specific surface and, therefore, loading efficiency of ibuprofen (an anti-inflammatory drug) was observed [88]. The release efficiency of gentamicin-loaded anodic TiO_2_ was compared for both nanotubes with and without a 2.5 μm thick coat of chitosan and poly(lactic-co-glycolic acid) (PLGA) [89]. In contrast to uncoated NPs that release the drug after 2 weeks, the modified nanooxide demonstrated extended-release up to 22 and 26 days for chitosan and PLGA, respectively. In 2018, Masoudi et al. [90] prepared TiO_2_ NPs with inherited fluorescence properties and high doxorubicin hydrochloride (DOX) loading capacity. In vitro cytotoxicity test on human osteosarcoma (SaOs-2) and breast cancer (MCF-7) cell lines revealed higher anticancer efficacy (lowered IC50 concentration by 5.5- and 3 fold for MCF-7 and SaOs-2, respectively) and better imaging for intercellular tracking of DOX loaded NPs relative to free DOX. In another study, diamond-shaped TiO_2_ NPs were functionalized with PEG chains and loaded with DOX [91]. In acidic conditions associated with cancer cells, DOX was almost entirely released. During in vivo tests, Bulb/c mice bearing H22 tumors showed smaller tumor volumes when treated with the complex drug nanocarrier as compared with DOX-free treated groups.

It follows that MONPs in combination with target molecules and drugs offer an efficient platform for delivery of therapeutics thanks to either simple delivery or other active release mechanisms. In that way, fewer adverse effects in health cells occur while the amount of the therapeutic agent is significantly lower. By assessment of a mixture of different drugs and therapies encouraging results have been reported.

### 3.2. Immuno-Therapy

NPs can interact with different components of the immune system and thus enhance or inhibit its function [92]. NPs can be specifically designed to suppress (for example anti-inflammatory) and stimulate (i.e. vaccine) immunity. The first step of recognition of NPs by the immune system as foreign materials is the adsorption of blood proteins which type and quantity determine the fate of NPs by interaction with other molecules. If NPs bind to the cell surface, they can initiate signaling processes triggering immunogenicity or toxicity. After activation, the immune system can release cytokines that act as a mediator of systemic and local inflammatory and hypersensitive reactions [11]. For instance, since ROS can function as a second messenger and modulator in immunity, ROS from TiO_2_ NPs can activate downstream pro-inflammatory effects and avoid the innate immunity in macrophages [37]. These observations suggest that MONPs with different surface reactivity can modulate the immune function trough ROS-activated pathways. Attachment or encapsulation of charged molecules such as enzymes, amino acids and so forth to NPs, can protect them from recognition by the immune system and prevent the adsorption of opsonins. Besides, careful design of various components can reduce the toxicity of these formulations [93].

#### 3.2.1. Iron Oxides Nanoparticles

Iron oxide NPs are also used as potent carriers for vaccine delivery with improved therapeutic effects. A way used to achieve successful inhibition of viral replication was the incorporation of small interfering RNA (siRNA) onto nanocarriers [94]. Other strategies include the use of transactivator or transcription protein as an adjuvant to target cell for intercellular delivery of MONPs or MONPs inhibition of viral glycoproteins that are vital to attach the host cell and thus inhibit the infection [95].

Iron oxide NPs were used as undercover carriers to deliver anti-retroviral drugs to a latent form of HIV while reporting the localization of drugs owing to their contrasting properties [96]. A complex anti-HIV drug, enfuvirtide that is unable to cross the blood-brain barrier (BBB), was able to cross it when the drug was loaded on a PMA amphiphilic polymer-coated over iron oxide NPs [97]. NPs have been shown to stimulate an immune response, including activation of antigen-presenting cells and induction of cytokine and chemokine release [98]. When captured by macrophages, supermagnetic iron oxide NPs could promote the pro-inflammatory phenotype in macrophages [99]. Shevtsov et al. [100] invented nanovaccine of superparamagnetic iron oxide NPs coated with recombinant heat shock protein 70 (Hsp 70) antigenic peptide that was able to stimulate tumor-specific T cell response in glioma bearing rats. By facilitating antigen trafficking to antigen-presenting cells (APC), a delayed tumor progression and increased overall survival were observed. In M2-polarised macrophages that are principally involved in wound healing, resolving inflammation, angiogenesis and tissue remodeling, internalized iron oxide NPs coated with dimercaptosuccinic acid, aminopropyl silane or aminodextran (the later as a prospective contrast agent and biomolecule delivery) showed no cell toxicity [52].

MONPs are also used to enhance the protective and long-lasting immune response of the safer but less immunogenic subunit vaccine than live attenuated vaccines. Citrate-coated MnFe_2_O_4_ NPs with protein corona of the fusion protein (CMX) composed of *Mycobacterium tuberculosis* antigens were proved to be capable of aiding the generation of the specific cellular immune response (T-helper 1, T-helper 17 and TCD8) [101]. Besides bacteria antigens, Fe_2_O_3_ NPs have been utilized in the creation of virus-like particles (VLP) that proved their exceptional use as vaccines, gene-carrying nanocontainers, MRI contrast agents and drug delivery vectors [102]. In this approach, bacterial viruses had been used not only as phages conjugated with functionalized (with carboxyl and amino groups, respectively, for better loading) MONPs for antimicrobial purposes [103] but also as a scaffold to carry more Fe_2_O_3_ NPs that can powerfully bind with cancer cell surfaces than Fe_2_O_3_ NPs themselves [104]. Moreover, antibody and antibody-like conjugates to NPs can be bi-specific complexes with multiple modes of action including the delivery of toxins or agents that kill tumor cells; inhibition of two ligands or receptors; and crossing of two receptors [105]. However, there are many gaps in understanding the immune response that MONPs induce.

#### 3.2.2. Zinc Oxide Nanoparticles

ZnO NPs have been reported to trigger cytokine and chemokine production which are used as biomarkers for immunotoxicity. They were found to exhibit adjuvant outcomes through diverse mechanisms including activation of the innate immune response, augmentation of the antigen uptake by antigen-presenting cells and regulation of cytokine network [44]. For instance, exposure of dendritic cells to ZnO NPs with negative zeta potential was found to upregulate the expression of CD80 and CD86 and stimulate the release of IL-6 and TNF-α without any cytotoxic effects [106]. Additionally, ZnO NPs were found to induce the degradation of IκBα that is NFκB inhibitor and thus to stimulate NFκB signaling [107]. Specifically designed ZnO tetrapod NPs demonstrated the effective suppressive function of HSV-2 genital infection in female Balb/c mice when used intravaginally [108]. The prior incubation of HSV-2 with ZnO NPs promoted local immune response similar to the infection but with less clinical manifestations and inflammation. The suppressed infectivity the authors attribute to facilitate intracellular delivery of viral antigen into APCs and, therefore, activated antigen-specific adaptive immunity.

Simultaneously, chemically synthesized NPs were found to be more toxic to cells and living tissue, whereas green synthesized NPs showed lesser toxicity probably because of phytochemical capping that may suppress the production of ROS [109] and thus the inflammatory reactions. Thatoi et al. [110] evaluated the anti-inflammation activity of bio-reduction synthesized ZnO NPs from mangrove plants namely *Heritiera fomes* and *Sonneratia apetala* by the capability of different NPs to impede protein denaturation trough blocking heat-induced albumin denaturation. The maximum inhibition activity (63.26 μg mL^−1^; IC_50_) was observed for *S. apetala* synthesized ZnO NPs followed by *H. fomes* synthesized ZnO NPs (72.35 μg mL^−1^ IC_50_) which values were close to that of conventional drug diclofenac (61.37 μg mL^−1^ IC_50_). In a dendritic cell line, PLLA microfibers coated with ZnO nanowires were observed to significantly induce inflammatory cytokines like IL-10, IL-6, IL-1β and TNF-α while introducing a tumor antigen [111]. The nanocarrier including ZnO enhanced the infiltration of T cells into the tumor tissue compared to mice immunized with PLLA fibers directly conjugated with tumor antigen. Recently, Hu et al. [112] showed an enhanced antitumor therapeutic effect in in vivo animal models injected with 4T1 tumor cells, especially in a combinatorial administration with ZnO NPs and doxorubicin (DOX). It was demonstrated that ZnO NPs promoted Atg5–regulated autophagy flux suggesting that autophagy induction by ZnO reinforces cancer cell death by increased Zn ion release and ROS generation when combined with DOX. It can be summarized that the use of MONPs increases the effectiveness of immunotherapy because of the specific characteristics of these nanocarrier systems. The targeting ability of NPs can be changed by different modifications including therapeutic agents, bioactive moieties or drugs. The research on the activities and effects of these NP systems is widely studied and still ongoing. Combined immunotherapy with other treatment methods such as chemotherapy, photothermal therapy and so forth, also gained attention. However, challenges remain due to lack of understanding about in vivo behavior of NPs that can act both as immune-stimulating adjuvants and/or delivery systems to activate antigen processing.

### 3.3. Diagnosis

MONPs have been extensively used for diagnostic purposes due to their fluorescent or magnetic behavior. The advantages of nano-oxides allow a more precise visualization and reliable quantification of disease viability and progression.

#### 3.3.1. Quantum Dots for Labeling

Various biomedical targets such as cancer cells, stem cells, bacteria or individual molecules could be labeled with highly fluorescent NPs. Quantum dots (QD) are highly fluorescent and photostable colloidal semiconductor NPs with 2–10 nm diameter [113] that is a useful tool in in vivo imaging cellular structures and events. In such a way monitoring of cell migration, retention in target sites or evaluation of viability can be done. When excited, QDs emit light with sharp and symmetrical emission spectra and high quantum yield. Their main characteristics are chemical and photostability, catalytic properties and controllable electron/ion transfer effect. However, the safe use of these QDs when introduced in the organism is of prime importance. For that reason, Wierzbinski et al. [114] investigated iron oxide NPs used as labeling agents for in vitro investigations of human skeleton myoblast cell line and in experimental animals after intramuscular and intracardial administration. The study showed no impact of labeling myoblasts with dimercaptosuccinate (DMSA) coated iron oxide NPs on their differentiation comparing to non-labeled cells. The gene expression levels of FTL (a biomarker for aging) encoding the light chain of the transferrin receptor that transfers the extracellular iron ions into the cytosol, was similar for both treated and untreated cells. Although influencing some gene expression, DMSA-coating NPs did not disturb stem cell homeostasis. Other authors improved cell labeling functionalization of ZnO NPs by applying silica coating which was conjugated with biotin (vitamin) amino group [115]. When avidin-attached nerve cells were exposed to biotin-conjugated ZnO NPs quantum dots with a size of 125 nm, green emission from the cells was observed and no emission was observed from silica-coated ZnO NPs without biotin. Additionally, biomarkers of NPs like fluorescent dyes were used for in vivo ocular imaging [116]. Further research should be done to elucidate the individual QDs transport, interactions, toxicology and fate in certain tissues, organs and organisms.

#### 3.3.2. Contrast Agents for Magnetic Resonance Imaging

Magnetic resonance imaging (MRI) is a widely used non-invasive clinical practice due to its good soft-tissue contrast, high spatial resolution, 3D anatomical information and non-ionizing radiation. Magnetic imaging techniques permit different molecular changes related to the inception and progress of pathological states to be counted and to provide timely diagnosis and prognosis of diseases such as cancer [117]. The contrast agents used in magnetic resonance imaging (MRI) are generally based on either iron oxide NPs or ferrites. Polymers are the most extensively used stabilizing materials for iron oxide NPs. Together they form negatively imaging agents producing a signal-decreasing effect and the persistent effects from circulating NPs delay MRI by 24–72 hours. When cancer cells of solid tumors are examined, in contrast to other tissues, the poor lymphatic drainage aids the entrapment of NPs. To increase signal sensitivity and enhance MRI diagnostics [118], alloy-based nanomaterials forming compounds known as ferrites (for example Zn-ferrite) that increase the net magnetization of NPs [119], were also used. Additionally, magnetoliposomes consisting of single iron oxide NP, vesicles of water-dispersible iron oxides or PEG encapsulated iron oxides core and covering single phospholipid bilayer [120] could increase the imaging contrast due to particle monodispersity. The ability to combine iron oxide NPs with other metals or to form core-shell iron oxide NP structures helps in improving the diagnostic information.

### 3.4. Nano-Oxides in Dentistry

Eliminating the bacterial infection in the root canal system is the decisive goal of endodontic treatment, preventing microorganisms from impairing periapical healing. For that reason, Javidi et al. [121] examined ZnO NPs for antibacterial root canal sealer by microleakage measurements at 3, 45 and 90 days intervals to check their stability and sealing properties. The microleakage increased with an increase in the calcination temperature (from 500 to 700 °C) because of an enlargement in the size of NPs and therefore, decreased effective surface. All prepared groups of ZnO NPs samples exhibited less microleakage in comparison with commonly used ZOE sealer. Another research group [122] synthesized more porous and spongier ZnO/MgO NPs for polycarboxylate dental cement used in clinical dentistry for luting agents, orthodontic attachments, cavity lining and bases and restoration for teeth. In contrast to conventional zinc polycarboxylate cement, ZnO and MgO nanostructured and ZnO/MgO nanocomposite cement had increased mechanical strength and comparable setting time for preparation of the dentin cement to those obtained by commercial samples.

Antibacterial nanofilms deposited on nanostructured electrochemically deposited TiO_2_ NPs on the surface of NiTi arches were evaluated in vivo for their histopathological, genotoxic and cytotoxic effects in Long-Evans rat [48]. After deposition, the coating was susceptible to degradation in PBS solution when ingests were absorbed and TiO_2_ NPs entered the bloodstream producing a toxic effect on the organs with the first step being the liver parenchyma which resulted in extensive lesions. When internalized, TiO_2_ NPs accumulated in the lysosomes which led to rupture and release of their content that subsequently activate apoptotic caspase pathways.

To generate novel biomaterial aimed at denture base that could obstruct the adhesion of microorganisms to their surface and therefore, to decrease the growth of denture stomatitis, Cierech et al. [123] modified polymethyl methacrylate (PMMA) with ZnO NPs. The increased hydrophilicity and hardness with absorbability explained the reduced microorganisms’ growth on the denture base. The antifungal properties increased with increasing the content of ZnO NPs in the polymer. Similarly, a thin layer of nanostructured TiO_2_ film of the surface of Co-Cr alloy revealed significant antifungal activity under UV-irradiation which can be considered in the future against denture stomatitis [124]. Nanotechnology offers enhanced effectiveness of already available technologies since it will be cost and time saving whilepreventing the patient from adverse side effects.

### 3.5. Nano-Oxides in Hard Tissue Regeneration

The bioactivity of the conventional titanium-based materials applied in orthopedic and dental implants is insufficient in terms of bone-implant osseointegration. It is widely known that the osseointegration of implants was enhanced if the surface topography and morphology of the natural tissue mimicked by implant surface. Most of the extracellular protein, structures and cell sizes vary between 10 to 100 nm. However, the implant material is not only recognized by targeting adherent cells but also from the host immune system that triggers immune reactions which may eventually determine the implant life. To increase cytocompatibility and decrease the macrophage activity, Li et al. [125] synthesized magnetron deposited cerium oxide NPs on titanium surface and examined the biological response and the underlying mechanisms of new bone formation in vitro and in vivo. The prepared oxide NPs were in mixed Ce^3+^/Ce^4+^ valence state and the increase in the surface Ce^3+^/Ce^4+^ ratio promoted new bone formation and mineralization. The lower surface Ce^3+^/Ce^4+^ ratio had high superoxide dismutase (SOD) and low catalase mimetic activities that regulated the production of ROS. Additionally, the surface Ce^3+^/Ce^4+^ ratio was found to modulate the balance of anti-inflammatory and pro-inflammatory cytokines in macrophages. It was concluded that the mixed-valence cerium oxide NPs had the potential to induce bone regeneration without the need of any exogenous osteogenic inducer. Even in the absence of osteogenic supplements, nanocomposite scaffolds of glass foams containing nanoceria demonstrated enhanced collagen production and osteoblastic differentiation of human mesenchymal stem cells (HMSCs) compared to scaffolds without nanoceria [126]. The authors assigned the enhanced production of collagen of HMSCs to the incorporation of nanoceria that acted as an oxygen buffer regulating the differentiation of HMSCs. However, the effects of the released cerium oxide NPs in the body are still unknown.

**Figure 5:**
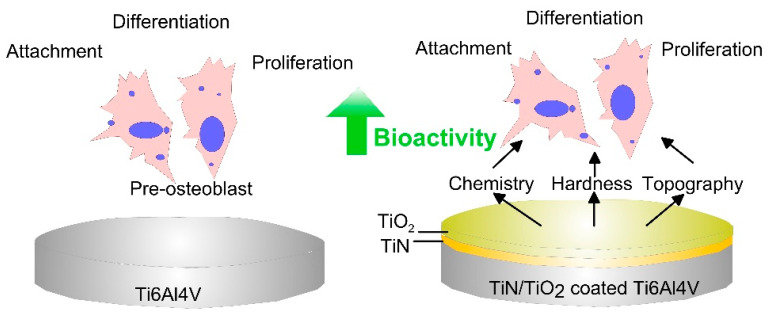
A strategy to improve osteointegration by applying inorganic nanostructured TiN/TiO_2_ coatings with enhanced surface characteristics as opposed to bare Ti6Al4V alloy.

TiO_2_ is extensively used for dental and orthopedic applications because of its biocompatibility and good mechanical properties. The morphofunctional reactions of the immortalized line of human T lymphocyte cells (Jurkat leukemia cells) to short-term in vitro exposure to rough micro-arc oxidated nano TiO_2_ coatings were examined by Kan Hlusov et al., in 2018 [127]. The metal-ceramic TiO_2_ film did not prompt apoptosis or necrosis in T-cells. However, the cells exposed to TiO_2_ coating with surface roughness value R_a_ > 2.2 μm exhibited a progressive decrease in viability and total suppression of IL-4 dependent mechanism of T-cell survival. The roughness of TiO_2_ dielectric surface-induced electrostatic potential, which was capable of varying the molecular genetic features and viability of leukemia T-cells via mechanisms discrete to ROS generation. Our research group found out that electron beam surface treatment of titanium alloy not only increased the surface micro- and nano roughness [128] but also decreased the surface hardness of the magnetron sputtered TiN with overlaying TiO_2_ coating bringing it closer to that of trabecular bone and human teeth and decreasing the elastic modulus mismatch between the bone and implant [129]. Moreover, nanostructured arc PVD deposited TiN with overlaying glow-plasma discharge deposited TiO_2_ coating indicated an increase in MG63 cell viability by almost 100% after 24 h relative to the bare Ti6Al4V alloys [130]. The coated samples displayed better cell attachment and cytocompatibility with no negative effects on MG63 cells (Figure 5).

Similarly, TiO_2_ nanotubes encourage interaction with osteoblasts by enhancing the contact area of the bone cell-implant. TiO_2_ nanotubes with diameters 30, 70 and 100 nm respectively, were investigated in vivo and compared with machined titanium implants for their bone-implant contact and osteogenic gene expression levels [131]. The optimum size of the nanotubes for rapid osseointegration was found to be 70 nm in diameter probably because of its similarity with the nanostructure of natural bone. It follows that by altering nanoscale dimensions, nanotubes were able to control cell fate and interfacial osteogenesis. Moreover, after comparing the bone mineral density and the volume of newly shaped bone, those around the propolis modified TiO_2_ nanotubes with a diameter of 60–90 nm were much higher than formed around individual TiO_2_ nanotubes at 1, 2, 3 and 4 weeks after implantation [53]. Collagen that is synthesized by differentiated osteoblast was found to be well-formed around the propolis-modified nanotubes. The expression of inflammatory cytokines (TNF-α) displayed the complete tissue around discrete TiO_2_ nanotubes but partially expressed around propolis modified TiO_2_ nanotubes. It is summarized that the identification of the influence of the specific surface features of biomaterials influencing the cell adhesion, proliferation or differentiation is difficult to be defined because of the complexity of the surface factors and cell mechanisms influencing their interaction.

### 3.6. Nano-Oxides for Wound Healing

MONPs are used not only in hard tissue reconstruction but also in polymeric nanofibers to enhance the overall properties of the composite scaffold for skin tissue engineering as wound dressing materials. Wound healing follows a complex sequence of events including enhanced accumulation of growth factors, proliferation of fibroblasts, and extracellular matrix synthesis that leads to re-epithelization. It has been proved that ZnO NPs cannot pass through the skin [132] and slowly solve in an aqueous solution as ions [133]. However, ROS generated by MONPs play signaling and regulatory roles in tissue engineering. It was found out that ROS assessed not only an antiseptic role in wound healing but they also participated in wound-to-leucocyte signaling in tissue [134]. For that reason, Augustine et al. [135] investigated the effect of ZnO NPs concentration incorporated in electrospun polycaprolactone (PCL) non-woven membrane on the cell proliferation of adult goat fibroblast cells. The results pointed out that the PLC membranes containing 0.5 and 1% ZnO NPs enhanced cell proliferation more than neat PLC-membrane. Another study also reported good fibroblasts proliferation results when ZnO NPs with concentration up to 1% was incorporated in PMMA fibers and films [136]. Recent studies demonstrated that the in vivo used microporous Ag/ZnO NPs loaded with chitosan accelerate significantly the initial state of the wound healing process in mice [137]. The composite dressings showed a long time moisture retention, enhanced blood clotting capability, high antibacterial activity against pathogenic bacteria and denser collagen deposition properties compared with either pure chitosan dressing or ZnO ointment gauze. Because not only ZnO NPs but also TiO_2_ NPs are proven to participate in increased ROS production in bioenvironment, they were recently used in novel cutaneous would treatments. TiO_2_-impregnated biodegradable zein-polydopamine-based nano-fibrous scaffolds demonstrated better-quality adhesion, proliferation and relocation of cells during in vitro tests [138]. Bacterial cellulose (BC–temporary skin substitute) loaded TiO_2_ nanocomposites (with inherent antimicrobial activity) were proved to participate in healing progression forming healthy granulation tissue and re-epithelization in deep partial-thickness burns in case of mice model [139]. BC-TiO_2_ nanocomposite treatment promoted suitable healing through fibroblast migration and suitable growth of epithelial cells along with blood supply restoration forming new blood vessels.

### 3.7. Nano-Oxides Used as Biosensors

**Figure 6:**
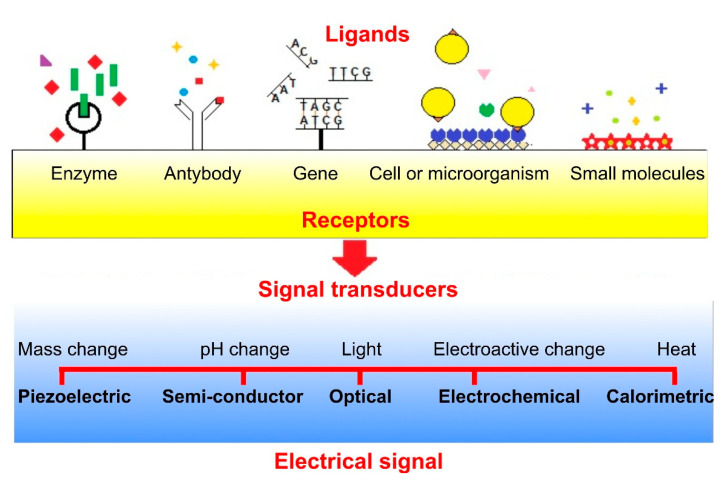
The illustration represents different mechanisms of biosensor activity; these bioreactions are carried out in a controlled environment on the immobilization platform. Depending on the generated signal, different transducing platforms are utilized to convert it into an electrical signal.

Nanobiosensors base on a ligand-receptor binding that evokes a reaction in the signal transducer. According to their detection principle (signal measuring), some authors [140] classify the nanobiosensors to piezoelectric, electrochemical, semiconductor, optical and calorimetric and all of them transferred the information into electrical signals. The electrode material is crucial in the fabrication of high-performance electrochemical sensing platforms detecting target molecules using different advanced analytical principles [141]. The nanobiosensors can be enzyme-based, genosensors, immunosensors, cytosensors, and biosensors for the detection of small molecules. The main mechanisms of biosensing are presented in Figure 6.

**Small molecule electroanalysis** is used to determine the presence of H_2_O_2_, glucose or dopamine. For example, α-Fe_2_O_3_ NPs in the form of cubes synthesized of hydrophobic iron-containing liquid under hydrothermal conditions was used as a glucose biosensor material for non-enzyme catalytic oxidation with high sensitivity and fast response [142]. MO nanostructures have been widely used in the immobilization of enzymes because of strong adsorption capability, enhanced electron-transfer kinetics and improved biosensing characteristics [143]. The **enzyme-based biosensors** contain a thin layer of the immobilized enzyme on the surface of the working electrode. For instance, the immobilization of the enzyme lactase detecting dopamine depends on the morphology of NPs used [144]. Comparing three kinds of phytic acid modified silica NPs with different morphologies; spherical, rod-like, and helical, for efficiency in dopamine detection, the best electrochemical performance was defined for the helical-shaped NPs. TiO_2_ NPs on silica sol-gel modified gold electrode immobilized with the enzyme lactate dehydrogenase were used as a biosensor for determining lactic acid with a detection limit of 0.4 μmol L^−1^ and small Michaelis–Menten constant (K_m_^app^ = 2.2 μmol L^−1^) [145]. Because of the large surface area and biocompatibility, ZnO nanorods functionalized with glucose oxidase enzyme have been exploited as a miniaturized biosensor for intracellular glucose measurements [146]. These nanostructured electrodes offer a biocompatible and electroactive surface for enzyme immobilization with enhanced orientation and biological activity [147]. If the nanostructure is porous the active surface for protein binding increases and it provides a protective environment for the enzyme to retain its activity and stability [148].

In the **genosensors**, single strained DNA fragments are immobilized on the electrode surface while the immunosensors are based on the specific antigen-antibody recognition. The conventional DNA detection techniques mostly use fast electrochemical biosensors because of their high specificity, portability and low cost [149]. The use of nanostructured materials allows for reducing the size, reagent and sample consumption and development of a microfluidic genosensing system with increased sensitivity. For example, CeO_2_ nanoshuttles-carbon nanotubes showed a remarkable synergistic effect enhancing the immobilization of DNA and, therefore, enhancing the sensitivity of target DNA detection via hybridization [150]. Another work combined the strong adsorption ability of Fe_2_O_3_ microspheres to DNA and the excellent conductivity of self-doped polyaniline nanofibers (SPAN) on carbon ionic liquid electrode for electrochemical impedance sensing of immobilization and hybridization of DNA. The proposed approach had a wide detection range, low detection limit (2.1 × 10^−14^ mol L^−1^) and successfully discriminated against the target DNA from mismatch sequences [151].

The **immunosensors** employ either antibodies or antigens as bio-recognition elements usually in combination with electrochemical transducers [143]. The sensing total performance depends on the density of the immobilized antibodies/antigens which makes MONPs appropriate for improving the functionality of the sensor. ZrO_2_ NPs on reduced graphene oxide had been functionalized using 3-aminopropyl triethoxy saline (APTES), electrophoretically deposited on ITO coated glass and further biofunctionalized with proteinaceous biomarker CYFRA-21-1 secreted in higher concentration in oral cancer patients [152]. This immunosensor platform exhibited a wider linear detection range (2–22 ng mL^−1^), excellent sensitivity (0.756 μA mL ng^−1^) and low detection limit (0.122 ng mL^−1^). The shelf life of the immunoelectrode was found to be equal to 8 weeks. Upon immobilizing with rabbit-immunoglobulin antibodies and bovine serum on nanostructured CeO_2_ film onto ITO coated glass, fungal toxin-ochratoxin-A could be detected [153]. The immunoelectrode exhibit enhanced characteristics in the linear range (0.5–6 ng dL^−1^), low detection limit (0.25 ng dL^−1^), rapid response time (30 s), higher sensitivity (1.27 μA ng^−1^ dl^−1^ sm^−2^), and high value of the association constant (K_a_ = 0.9 × 10^11^ L mol^−1^). Gold/ZnO nanocomposite films were employed to enhance the performance of surface plasmon resonance (SPR) for tumor marker detection [154]. The linear range of response to carbohydrate antigen extended from 1 to 40 U mL^−1^ and the limit detection fell to 0.025 U mL^−1^. The charged intensity of Au/ZnO sensor was increased by nearly 2 folds of Au/Cr layers whereas the detection limit decrease 4 times than Au/Cr layers.

The **cytosensors** recognize antibodies, receptors, glycans or other molecules overexpressed on the cell membrane of a target cell. For example, TiO_2_ nanowires functionalized with monoclonal antibodies and immobilized on gold microelectrodes through mask welding were demonstrated as sensitive, specific and rapid in the determination of bacteria *Listeria monocytogenes* for concentrations at low levels of 102 cfu mL^−1^ for 1 hour without interfering with other food-borne pathogens [155].

The photoelectrochemical assays always have advantages of both optical and electrochemical detection [156]. The method could detect biomolecules such as glutathione at relatively very low applied potentials using porphyrin-functionalized TiO_2_ NPs. Similar porphyrin-functionalized ZnO NPs were advanced for the detection of cysteine with broad linearity in the range of 0.6–157 mmol L^−1^ in physiological media. The ability of nanobiosensors to be easily displaced and deformed in response to very low forces of about 10 pN makes them sensitive enough to detect even the breaking of individual hydrogen bonds [157]. It is clear that the key features of MONPs like high sensitivity and selectivity, fast response and recovery times, reversibility, integration in different scales make these substances suitable for the monitoring infection diseases, the pharmacokinetics of drugs, detecting of biomarkers (cancer and disease), small molecules and so forth.

### 3.8. Antimicrobial Nano-Oxides

**Figure 7:**
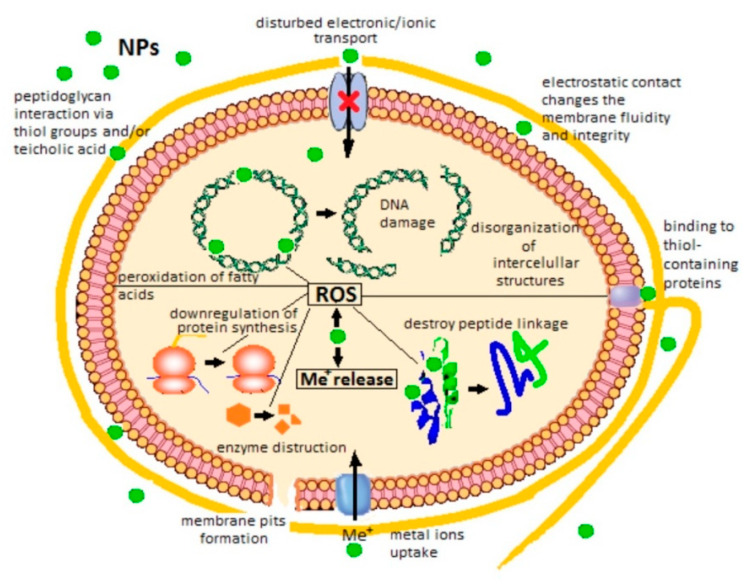
Mechanisms of bacteria cell damage by MONPs, illustrating the possible MONP interactions with the bacterial cell wall, membrane components, DNA, enzymes and other proteins and the influence of external ROS on membrane integrity, protein synthesis and functioning. Different MONPs may cause toxicity via one or more of the described mechanisms.

The interaction between NPs and bacteria usually triggers toxic effects that are exploited for antimicrobial applications in industries such as food or agriculture. On one hand, the increasing problem with AB resistance strains due to the transfer of AB resistance genes between bacteria could be overcome by using bactericidal NPs that substitute certain conventional antibiotics. On the other hand, the number of new fungal infections in immunocompromised patients and plants is increasing throughout the whole world. *Candida albicans* is the most common yeast colonizing the skin, reproductive system and gastrointestinal tract [158]. NPs interfere with different cellular processes of the pathogens and the emergence of resistance is less likely to occur [159]. The antibacterial activity of MONPs depends on mixture concentration and pH, size, distribution, and agglomeration of NPs and displays dose- and time-dependent efficiency. Yamamoto et al. [160] demonstrated that when the suspension of powder MO was concentrated enough, the larger the specific area, the better the antibacterial activity. Also, the increase of speed of agitation of the dispersion of NPs in suspension increases the death of bacteria indicating the importance of frequency of electrostatic contact and its intimacy between NPs and cells [161]. The factors that influence the sensitivity of the bacteria to oxide NPs are the synthesis parameters of NPs, structure of bacterial cell wall, and degree of contact with the bacterial cell [162]. Figure 7 summarizes the main mechanisms of bacterial cell damage triggered by the presence of MONPs.

NPs as antimicrobial agents are highly promising because they have numerous modes of action but the major lethal pathways usually occur simultaneously. It is generally accepted that most of the MONPs exhibit bactericidal properties by generating ROS or releasing metal ions. For example, the photocatalytic toxicity was found to induce lipid peroxidation under near UV-lamp causing respiratory dysfunction and death of *E. coli* [163] and when combining with other nanomaterials like Ag particles, synergetic antibacterial effects were observed [164]. NPs affect also the cell membrane potential and integrity [165] and change the metal ion uptake into the cells followed by depletion in adenosine triphosphate (ATP) production and DNA replication [166].

The modern alternative for the production of MONPs from environment-friendly, non-toxic and save reagents could use inactivated plant tissue, plant extracts, extrudates and other parts of the living plant organism. The biosynthesis involves reducing metal ions or oxides with the help of phytochemicals such as polysaccharides, reducing sugar, amino acids, alkaloids, vitamins, terpenoids, saponins and other plant substances [167]. Except for different metal salts and reducing agents, the biosynthesis of MONPs using plant extracts involves stabilizing or capping agents for better size control of MONPs, prevention of aggregation [168], and, therefore, improvement of the biological potential of NPs. Other organisms that are used for “green” methods of MONPs synthesis are bacteria, fungi, yeasts, and algae but the plant extract mediated biosynthesis was found to be more stable and faster than microbial synthesis [169]. The rate of synthesis of NPs was related to the reaction and incubation temperature with the following tendency: the higher the temperature, the faster the rate and the smaller the average size of the particles. The natural synthesis is environment-friendly, cost-effective, taking less time and does not require costly equipment and precursors [170]. Recent works focusing on the green synthesis of MONPs and their antibacterial effect are tabulated in Table 1. 

#### 3.8.1. Titanium Dioxide Nanoparticles

TiO_2_ in the anatase phase is shown to be the most potent form of producing ROS [199]. According to Cho et al. [200], superoxide alone played an insignificant role in the inactivation mechanism of bacteria in contrast to OH• radical which was primarily responsible for *E. coli* inactivation. The photooxidation decomposition and mineralization peroxidation of the bacterial plasma membrane saturated and unsaturated fatty acids are thought to be one of the reasons for high antibacterial activity [201]. Another study confirms that TiO_2_ NPs reacted with thiol (-SH) groups of the proteins in bacterial cell wall causing inactivation of transport proteins nutrients and reduction of cell permeability which triggered cell death [202]. Moreover, the bactericidal activity of UV-light-activated anatase TiO_2_ NPs was dependent on the amount of dissolved molecular oxygen and the proper cell-NPs contact that accelerated the translocation of NPs across the microbe cell wall [203]. TiO_2_ NPs photocatalysis showed different dysfunctions such as cell inactivation at the regulatory and signaling level, a decrease in coenzyme–independent respiratory chain, lower ability to transport and assimilate Fe and P, and lower capacity of heme biosynthesis and degradation [204]. With the addition of metals such as Pt, Au, Ag, Ni, and Cu to TiO_2_ NPs, the UV-light-activated bactericidal activity of nanocomposites against *E. coli* was found to be greater compared to pure TiO_2_ NPs [205]. Simultaneously, in the absence of photoactivation, TiO_2_ NPs were not losing their antimicrobial activity that could be explained with the electromagnetic attraction between the microorganisms and MONPs that induced oxidation reactions [206]. The antimicrobial activity of TiO_2_ NPs modified with *G. zeylanica* (endemic plant of Sri Lanka) aqueous extract was enhanced because of the multiple mechanisms of phytochemicals and when exposed to sunlight, the bactericidal activities of the modified TiO_2_ NPs were additionally improved [207]. Titania NPs showed inhibitory activity towards H9N2 avian influenza virus which activity increased under UV light [208]. TiO_2_ NPs tagged with DNA encoding region of the viral DNA was able to inhibit H5N1 and H1N1 viruses replication [209].

#### 3.8.2. Zinc Oxide Nanoparticles

ZnO NPs hold high optical absorption in UVA and UVB spectrum which property is important for their antibacterial response and protection of UV light in cosmetics. The bactericidal effect of ZnO against gram-negative [210] and gram-positive [211] bacteria was found to be dependent on the oxide concentration and size of MONPs [212]. As an n-type semiconductor, ZnO NPs in aqueous solution absorbed UV irradiation and showed phototoxic effect producing ROS like H_2_O_2_ and superoxide ions (O_2_^•−^) that could inhibit or kill microorganisms [213] by interacting with active enzymes, proteins, and DNA [214]. It is established that high atom density (111) facets of ZnO crystal lattice exhibited higher bactericidal activity [215]. The comparison of the antibacterial activity of ZnO NPs in dependence on the size of particles indicated the best bactericidal response of the particles with a smaller size [26] because of the concentration of oxygen species on the surface was higher for the higher surface area. TEM analysis of *S. aureus* and *B. cereus* membranes showed that ZnO NPs caused bacteria cell deformation and damaging together with disorganization of the intercellular structures [216]. Some authors determined that the antibacterial activity did not correlate with ROS levels or Zn^2+^ ion release [217] while others explained the damage of cell membranes with creating electrostatic forces between the negatively charged cell surfaces and ZnO NPs containing positive charges in water suspension [218]. ZnO NPs mediated killing of *S. aureus* caused significant up-regulation of pyrimidine biosynthesis and carbohydrate degradation while the amino acid synthesis was down-regulated [219]. ZnO NPs showed not only antibacterial but also antifungal activity against *A. invadans* at a concentration lower than that of silver NPs against the same fungus while simultaneously caused higher cytotoxicity [220]. Because of the attraction of negatively charged ZnO NPs towards viruses, ZnO tetrapod type structures were able to control and trap both herpes simplex virus HSV-1 and HSV-2 [221,222] which make them of specific interest in prophylactic therapy and/or maintaining latency.

#### 3.8.3. Copper Oxide Nanoparticles

CuO NPs are far cheaper than AgO NPs but higher concentrations are required to obtain the anticipated antimicrobial effect. It is believed that the microbial toxicity of CuO NPs is mainly due to Cu^2+^ ion release [223,224]. CuO NPs are more effective towards bacterial species with cell walls rich in amine and carboxyl groups such as gram-positive *B. subtilis* [225]. Since the multilayered peptidoglycans in gram-positive strains are negatively charged, they can bind Cu^2+^ ions released from CuO NPs. At pH 6 and 7, gram-positive *S. aureus* strains were more resistant to CuO NPs as opposed to pH 5 where the higher toxicity of CuO NPs was related to increased release of Cu^2+^ and induced ROS response [226]. The smaller CuO NPs with a size of around 20 nm appeared to have strong bactericidal activity against both gram-positive and gram-negative bacteria [227]. Concerning gram-negative *E. coli*, Cu_2_O (cubic) NPs were more efficient against the microorganisms than CuO (monoclinic) showing higher affinity to the bacterial cell while CuO NPs produced significant ROS in terms of superoxides than Cu_2_O [228]. At concentration 50 μg mL^−1^, CuO NPs had higher biocidal activity against microorganisms of oral microbiota than ZnO NPs [229] without showing genotoxic or cytotoxic effects on cancerous HeLa cells in the same doses [230]. CuO NPs did not have M13 bacteriophage inactivation activity, whereas dual photoexcitation by UV irradiation-induced not only bactericidal but significant anti-phage activity [231]. Besides, the multiple responses of *P. aeruginosa* exposed to CuO NPs induced lysogenic bacteriophage which might render defective within the bacterial host such as nitride accumulation increased N_2_O emission and inhibited respiration [232].

#### 3.8.4. Silver Oxide Nanoparticles

Silver has a beneficial effect to be toxic even at low concentrations against bacteria [233]. It is generally accepted that highly reactive silver ions lyse or ultimately kill the bacteria cells. Similarly, silver is known to interact with the thiol groups (-SH) of proteins or generate ROS which contributes to the antimicrobial properties [204,205,206,207,208,209,210,211,212,213,214,215,216,217,218,219,220,221,222,223,224,225,226,227,228,229,230,231,232,233,234]. The accumulation of silver NPs in microbial membranes was shown to cause increased permeability and finally, the membrane was damaged by the free radicals formed by the presence of NPs [235]. The bactericidal activity of AgO NPs produced by microbial cultures was almost the same as that of silver NPs and they were effective against both gram-positive cocci and rods [236]. The hemolysis effect of Ag_2_O NPs prepared either by chemical or green methods with similar sizes (~30 nm) and almost the same spherical shape was found to be highly different [237] from Ag NPs that were stable in nature and had lower hemolysis effect. The oxide NPs showed an increase in lysis properties due to redox processes triggering interfacial charge interactions. The comparison of functionalized with 5-amino-2-mercapto benzimidazole Ag_2_O_3_ NPs with non-functionalized, demonstrated that antimicrobial activity against *S. aureus* and *P. aeruginosa* of the functionalized NPs was decreased [238]. Additionally, Ag_2_O_3_ NPs demonstrated significantly toxic effects against the fungus *A. niger* in contrast to the modified NPs. The 5-amino-2-mercaptobenzimidazole did not have antibacterial behavior against gram-positive but possessed excellent bactericidal effect against gram-negative. The authors explained the reduced bactericidal activity of the functionalized NPs with the reduced interaction of Ag_2_O_3_ NPs with the cell membrane and a slight increase in particle size.

#### 3.8.5. Magnesium Oxide Nanoparticles

MgO NPs trigger post activation of the bone-repair scaffold and are used as hyperthermia agents in cancer therapy [239]. MgO NPs exhibited effective bactericidal potential against both gram-positive (*S. aureus*) and gram-negative (*E. coli*) bacteria [240] and even against fungi such as *Aspergillus niger* and *Penicillium oxalicum* [241]. MgO NPs demonstrated better inhibition for rod-shaped bacteria (*E. coli*) in contrast to spherical-shaped bacteria [242]. Portions of MgO NPs possibly react with water to form Mg(OH)_2_ which could lose OH^−^ ion into the solutionand increase the pH. It was previously demonstrated that the peptide linkage in the cell membrane of *Pseudomonas aeruginosa* and *E. coli* was destroyed by the generated superoxide ions on the surface of MgO NPs [243]. O_2_^−^ was more stable in the alkaline environment that contributed to the higher antibacterial effect of MgO NPs [244]. The mean zeta-potential of MgO NPs exhibits a positive charge in pH range from 4 to 8 [245] favoring the electrostatic interaction of NPs with bacteria cells. These MgO NPs could completely kill phytopathogen bacteria *R. solanacearum* at a comparatively higher concentration, 250 μg mL^−1^. Similarly to the other MONPs, MgO NPs destroyed and disintegrated the cell wall of the phytopathogen bacteria leading to leakage of the intercellular content and cell death. The same authors discovered that except for retained biofilm formation, MgO NPs improved the bacterial susceptibility to antibiotics. In contrast to nisin (antibiotic) addition, MgO and ZrO mixing did not enhance MgO activity against pathogens [246]. MgO NPs possess also the advantage of not being cytotoxic to human cells at lower concentrations (0.3 mg mL^−1^) [247]. However, the bactericidal activity of MgO NPs increases with raising the concentration of NPs.

#### 3.8.6. Calcium Oxide Nanoparticles

Similarly to MgO NPs, the formation of ROS in the presence of CaO NPs was influenced by the higher pH that helped in antibacterial activity [248]. For both MgO and CaO, the contact between NPs and bacteria is an important factor for the bactericidal activity [249]. The active superoxides ions reacted with the carbonyl group in the peptide linkages of the cell wall of bacteria and disrupted them [250]. High-concentrated CaO and MgO NPs slurries in physiological saline were even able to kill spores of *B. subtilis* that are more robust with their thick proteinous wall than the vegetative cells. In contrast to the little affinity of ZnO NPs to fungal cells, the alkali MONPs – CaO and MgO, showed stronger affinity to postharvest fungal cells (*Saccharomyces cerevisiae*, *Candida albicans*, *Aspergillus niger* and *Rhizopus stolonifer*) than to bacterial cells [251]. This suggests that the yeasts were killed by the high pH of the alkali oxides because of the solubilization of cell surface proteins and the presence of alkali-soluble polysaccharides [252].

#### 3.8.7. Aluminum Oxide Nanoparticles

It was found that alumina NPs worked against *E. coli* only at high concentrations [253]. Al_2_O_3_ NPs synthesized by co-precipitation method with irregular shape and size of around 35 nm demonstrated effective antibacterial activity not only against *E. coli* but also against *P. vulgaris, S. aureus* and *S. mutans* [254]. The highest was the susceptibility of *E. coli* and the smallest for *P. vulgaris*. Like the other oxide NPs, their interaction with the cell wall distorts the cell morphology. Ansari at al. [253] characterized the damage of the *E. coli* cell wall under the influence of Al_2_O_3_ NPs as the formation of pits. Agglomerated particles remained adherent to the outer surface while small-sized NPs were found uniformly distributed inside the cells. The lipopolysaccharides from the cell wall-bound to alumina NPs in a similar manner as the bacterial cell bound to a solid surface. It was proposed that the interaction between the amphiphilic biomolecules and Al_2_O_3_ NPs could affect the membrane fluidity and integrity explaining the antibiosis of NPs. Jiang et al. [255] examined whether the bactericidal activity of alumina NPs was due to the particles in the suspension or due to the ion release which could damage the cell membrane. It was established that there were no dissolved aluminum ions detected in the supernatant of suspension leading to the conclusion that the attachment of NPs to the cell wall was of prime importance for the antibacterial activity of alumina NPs. Alumina-silver composite NPs produced by the wet chemical method and modified with oleic acid as a capping agent demonstrated slightly higher antimicrobial activity against gram-positive *S. epidermidis* bacteria than against *E. coli* but both bacteria were more sensitive to the complex NPs as opposed to alumina NPs themselves [256].

#### 3.8.8. Iron Oxide Nanoparticles

The magnetite (Fe_3_O_4_) and maghemite (Fe_2_O_3_) indicate single crystalline in structure biocompatible materials showing superparamagnetic behavior. Due to the strong magnetic attraction among NPs and high energy surfaces, they tend to agglomerate. Combining Fe_2_O_3_ NPs with Ag NPs could avoid coalescence and contamination of the surrounding by achieving the recycling of nanosized silver [257]. The resultant heterodimer NPs base on magnetic core coated with ethylenediamine ((CH_2_)_2_(NH_2_)_2_) modified chitosan/polyacrylic acid (PAA) and Ag NPs demonstrated excellent antibacterial against both *S. aureus* and *E.coli* because of the electrostatic sorption to bacteria cell and the enhanced efficiency by Ag NPs. The iron oxide–magnetite, is of great interest because of high magnetism, biocompatibility, low cost and low toxicity [258]. Fe_3_O_4_ NPs synthesized by top-down pulse laser ablation in two types of solutions, SDS and DMF (dimethylformamide) indicated high biocidal action against both gram-positive (G+) and gram-negative (G−) bacteria [259]. The stress in G− bacteria the authors explained with the formation of ROS generated with the interplay of oxygen with reduced iron species (Fe^3+/^Fe^2+^) or disturbed electronic or ionic transport chains in the cell membrane. The higher Fe_2_O_3_ NPs effectiveness against G+ bacteria the authors related to the smaller negative charge of *S. aureus* membrane than that of *E. coli* causing a higher level of penetration of the negatively charged NPs. The high antimicrobial activity of iron oxide NPs synthesized using leaf extract of *Cynomtra ramiflora* against *E. coli* and especially against *S. epidermidis* attributed to the synergetic effect of the phytochemical residing in the leaf extract and ROS produced by NPs [260]. These results suggested a synergetic effect of the phytochemical alkaloids and generation of ROS by the oxide NPs.

#### 3.8.9. Nickel Oxide Nanoparticles

NiO NPs were found to have bactericidal and bacteriostatic activity depending on the bacterial species and concentration. We synthesized Nickel-based Gadolinia doped ceria with spherical shape and size between 40–70 nm with composition NiO-Ce_0.8_Gd_0.2_O_2-δ_ via co-precipitation [261]. At a concentration equal to 100 µg mL^−1^, the antibacterial activity of the prepared NiO-CGO nanocomposite against Gram-negative (*E. coli* and *V. cholerae*) strains was found to be slightly lower than that of standard antibiotic (Streptomycin). After surface functionalization, both the bactericidal activity (against *S. aureus* and *P. aeruginosa)* and antifungal activity of 5-amino-2-mercaptobenzimidazole NiO NPs were increased compared with the non-functionalized NiO NPs [262]. The authors explained the observed difference with the enhanced dispersibility of the modified NPs. As Ni ions could not pass through the cell membrane ion channels, small NPs must have been uptaken by the cells and extracellular Ni ions might interfere with intracellular Ca^2+^ metabolism, which might be the reason for cellular damage. NiO NPs are also shown to interact with the sulfur and phosphorous of the bacteria DNA and other functional groups of proteins, which led to protein leakage and bacteria death [263]. Green synthesized NiO NPs annealed at different temperatures showed moderate bactericidal activity toward suspension of microbial pathogens *S. aureus* and *E. coli* without completely inhibiting the cell growth of both strains [264].

#### 3.8.10. Cerium Dioxide Nanoparticles

In contrast to other NPs, CeO_2_ inhibits ROS rate of production, induced by H_2_O_2_ in a dose-dependent manner that suggests their high anti-oxidant potent [37]. CeO_2_ NPs showed activity against both the gram-positive and gram-negative bacteria but the greatest antimicrobial function was observed against gram-negative strains [265]. This is because CeO_2_ NPs are positively charged and easily bind to gram-negative bacteria by electrostatic attraction [266]. In contrast to other NPs, CeO_2_ NPs were not found to penetrate through the *E. coli* membrane [267]. The proposed mechanisms for bacteria inactivation were: electrostatic adsorption, modifying the cellular ion transport; oxi-reduction processes on the surface of NPs, resulting in oxidative stress on lipids and proteins of the plasma membrane; an attack on the electron-flow and bacterial respiration. CeO_2_ NPs synthesized by employing a solution combustion method with ceric ammonium nitrate and EDTA as fuel at 450 °C, demonstrated spherical form with an average size of 42 nm [268]. The authors found concentration-dependent bactericidal activity against *P. aeruginosa* and a lack of activity against gram-positive *S. aureus*. It was proposed that the reason for the observed effect was related to the presence of teichoic acid in the peptidoglycan layer of gram-negative bacteria that interact with CeO_2_ NPs. Recent examinations focus on the antimicrobial activity of Zr doped CeO_2_ NPs with different concentrations of Zr synthesized by precipitation method [269]. The varying ratio of Zr impregnated CeO_2_ NPs (5 to 15%) did not greatly influence the moderate antimicrobial activity against *S. aureus*, *E. coli*, *P. aeruginosa*, *S. faecalis*, *B. subtilis* and *P. vulgaris* of the modified NPs. It was also found out that these impregnated with Zr CeO_2_ NPs were not effective against pathogenic fungi like *C. albicans*, *A. terreus*, *C. tropicalis*, *A. flavus*, *A. fumigatus* and *A. niger.*

## 4. Nanotoxicology

Nanotechnology constitutes a very encouraging field for the production of new diagnostics and therapeutic agents with applications in medicine but merely a few nanoproducts were currently used in practice. The oxide NPs show good potential for therapy of different diseases and pathogen control. Besides the important biological functions of ROS such as cell proliferation, migration, signalization, wound healing and so forth, the generated by MONPs reactive species could produce not only an inflammatory response but generate oxidative stress above a certain amount and eventual apoptosis of normal cells. For example, Rizk et al. [47] demonstrated that the intoxication with TiO_2_ NPs caused complex multifactorial ailment routes, leading to chromosomal aberrations in bone marrow metaphase cells of intoxicated mice and surge in the activity of serum aspartate and alanine aminotransferase which is the markers designating severe inflammation and liver injury as a result of oxidative stress, relative to size and composition of NPs. Most of the researchers face with the issue of balance amongst the therapeutic or diagnostic effects of MONPs and their toxic side effects. The toxic effects are greatly dependent on the physicochemical properties of NPs, concentration, time of interaction, NPs’ stability in a certain environment, type of the metal oxides and cells tested, and MONPs’ accumulation in tissue and organs.

It now becomes clear that it is difficult to distinguish NPs-specific effects from the ionic effect that usually occur simultaneously. The ion dissolution is influenced by the type of NPs, their pH, ionic strength and interfering organic matter. Additional complexity is found when NPs tend to agglomerate. The low colloidal stability results in a decrease of NP concentration in the active media. Internalized in the body, NP aggregates were not broken up by biofluids and remained intact during distribution [270]. Moreover, NP aggregation that is affected by pH, temperature, ionic strength and so forth could change the behavior, mobility, bioavailability, and, therefore, the fate of the NPs in the organism. The interaction between NPs and macromolecules, cells, tissues and organs is critical in determining their behavior in biological systems. It has been postulated that the cell surface-NPs charge interaction enhanced the metabolic activity of the cells [271] but when the concentration of NPs increased to 300 μg mL^−1^ both proliferation and differentiation are hindered probably because the internalized NPs induce apoptosis [64]. NPs such as γ-Fe_2_O_3_ with no dose-dependent action demonstrated in vivo toxicity in rats due to biotic and abiotic dynamics and lack of target delivery. Moreover, 10 nm poly(ethylenimine)-coated iron oxide demonstrated in vivo dose-dependent lethal toxicity in Balb/c mice [12]. When examining the viability of mouse embryonic fibroblasts NIH3T3, Jarockyte et al [272] found that after 48 h of testing a dose-dependent decrease in cell viability is observed and only a low concentration of iron oxide NPs (32.5 ng mL^−1^) was completely eliminated within three weeks. Multicore iron oxide nanoflowers of around 140 nm administrated to a *Xenopus Laevis* animal model indicated that although the particles were not lethal in elevated doses, several malformations were observed in the embryos [273]. The genotoxicity and hematological changes of long-term (60 days) and low-dose (100 and 200 mg kg^−1^ b wt) oral exposure to anatase TiO_2_ NPs (5-10 nm) manifested swollen erythrocytes and macrocytic anemia while activation of the defense and immune system and inflammation occurred in the lungs, kidney, liver, and brain [274]. At the cell level, the effect of TiO_2_ NPs on human neuroblast cells (SH-SY5Y) indicated a lesser growth rate, greater shortening rate of microtubules, and condensed lifetimes of *de novo* synthesized microtubules [36,37,38,39,40,41,42,43,44,45,46,47,48,49,50,51]. In an interesting study, the toxicity of TiO_2_ nanowhiskers in combination with 5,10,15,20-tetra(sulfonatophenil)porphyrin (TSPP) assessed in vivo on rat was reduced as compared to pure TSPP especially at high concentrations [275]. According to MTT assay, the combination of 0.1 mM TSPP with 0.6 mM TiO_2_ revealed minimized cytotoxic effects because of the porous nature of NPs allowing a slow release of the adsorbed TSPP that helped in lowering the adverse effects.

Although Zn can be efficiently excreted from the body through urine, sweat, and feces, ZnO seems to cause an inflammatory response and in vivo toxicity [276]. When instilled into the respiratory tract, ZnO NPs induced lung inflammation by the production of pro-inflammatory cytokines via Toll-like receptor (TLR) signaling pathways in a myeloid differentiation primary response protein-88 dependent manner [277]. The intravenous injection of 2.4 mg kg^−1^ ZnO nanorods in mice model caused reduced platelet and blood cell counts, increased stress markers in the liver together with DNA damage in the spleen, liver, and kidney [278]. Injected at higher doses (30 mg kg^−1^) in rats, ZnO NPs triggered multifocal acute injuries in the lungs and elevated mitotic figures in hepatocytes [279]. Han et al. [280] reveal that the daily intraperitoneal injection of ZnO NPs to young Wistar rats over 8 weeks affected the functionality of the brain by enhancing the long-term potentiation while scarcely influencing the depotentiation in the dentate gyrus region of the hippocampus. This bidirectional effect of ZnO NPs on long-term synaptic plasticity broke the balance between stability and flexibility of cognition which could modulate the efficiency of spatial cognition. Consequently, before implementing toxicity assessment the particle size, distribution, composition, morphology, surface area and chemistry, intrinsic properties and particle reactivity in the solution should be taken into consideration for accurate characterization. If the adoption and succeeding distribution are highly irrelevant, the impending risk upon exposure can be limited to local and indirect effects, while the sluggish elimination or whole retention result in NPs buildup and increased internal exposure [270].

Another important parameter influencing the long term toxicity of MONPs is their biodegradation. Highly acidic media with certain proteins found in lysosomes can degrade the particle coatings and trigger NP corrosion [281]. By incubation for 24 hours with artificial lysosomal fluid (pH 4.5), ZnO NPs dissolved around 90%, whereas, in artificial interstitial fluid (pH 7.4), the particles showed no dissolution [282]. The elevated Zn^2+^ ion concentration increased intercellular ROS, DNA damage and triggered apoptotic cell death [283]. In the degradation of nanocube iron oxide NPs, the formation of ferritin in a murine model was observed proving the activation of iron metabolism routes that deal with iron coming from NP degradation [284]. The presence of coating and aggregation of NPs are critical parameters decreasing the degradation kinetics. Recently, it was described that in tumor cells, transitional metals like iron, copper and so forth contribute to regulated nonapoptotic cell death called ferroptosis brought by oxidative stress response and presenting no morphological or biochemical markers of cell death [285]. Ferroptosis exhibited many similarities to cell death called oxytosis such as the disintegration of intercellular organelles, glutathione depletion with loss of glutathione peroxidase 4 activity, ROS generation and an increase of cellular calcium. The reduced capacity of iron storage in serum ferritin complexes on one hand and on the other - the increase in iron uptake through either the membrane-bound transferrin receptor 1 (ThR1) or within endosome compartments, contribute to intercellular iron overload and subsequent generation of highly reactive hydroxyl radicals creating lipid hydroperoxides and production of lipid ROS [286]. Mitochondria, lysosomes, endoplasmic reticulum and cell membrane contributed to overall levels of ROS production and together with the increased cytoplasmic calcium, they could eventually trigger oxidative cell death. It follows that when NPs start degrading ferroptosis will have a negative effect but if new oxide-based NP constructs with controlled atom release are designed, they can be used as a positive tool in cancer therapy.

Following the tabulated studies, it is extremely hard to draw an unambiguous conclusion regarding the relevance of ion release and ROS from NPs to the various toxicological effects of the nanosized agents. NPs of the same chemical composition occurring in different nanoforms concerning size, shape, surface affinity and so forth, may exhibit different hazard and fate profiles [287]. Add to this, the combined exposure of MONPs with other chemical substances can trigger unexpected and contradictory results. Moreover, the impact of the coating or modifying shell is not a matter of indifference. The relative biological effects of NPs with different initial characteristics such as chemistry, size, shape and crystallinity which characteristics usually trigger mixture toxicity effects (such as protein binding, enzyme activation, different NP interactions, etc), induce different sensitivity while using different cytotoxic assays which may lead to diverging or wrong conclusions. Therefore, some of the common methods have to be upgraded and adapted and new ones together with test guidelines and regulatory frameworks for in vivo, in vitro, and/or in silico testing of NP toxicological effects have to be developed.

## 5. Summary and Future Prospectives

The primary goal of this review was to set a reference to researchers who are interested in nanoparticle-based functional biomedical applications. MONPs possess huge potential as substances fighting multidrug-resistant microorganisms that could substitute antibiotics. The redox-active MONPs tend to modulate the human innate and adaptive immunity and this ability could be successfully used in enhancing immune response during vaccination or mediating immune tolerance against autoimmune diseases, allergies or cancer. The application of MONPs as molecular imaging agents, drug vehicles, and cancer therapeutics is extremely promising but there are many challenges and emerging problems that need to be solved before clinical or industrial applications. Despite the huge potential of MONPs in the biomedical area, the major drawback of ROS-based therapy is the inability to control ROS delivery to tissues or cells. However, there are also certain unknown mechanisms of cellular and extracellular functioning of MONPs that are unrelated to ROS generation waiting to be successfully established.

MONPs were debated in detail for their application in nanomedicine as tissue and immunotherapeutics, quantum dots, in dentistry, regenerative medicine, wound healing, also as biosensors. Their antimicrobial, antifungal and antiviral properties were also well discussed. With a vast number of different metal oxide NPs, only the majority of examples could be mentioned here by emphasizing the principal advantages of such MONPs. MONPs have emerged as a new generation of materials that go further than conventional applications of bulk materials. The variety of unique properties makes them potentially of great use in fuel cells, energy storage and conversion, catalysis, solar cells, optical displays, sensors and many other electronic and optoelectronic devices [6].

Considering certain applications, the biological effects and cytotoxicity should be conferred concerning MONPs type, size, concentration, synthesis method, and so forth. This should also include in vivo examinations of the long-term health risks. Both advantages and disadvantages represent a challenge sharing material scientists and life scientists who ought to develop and examine the structure-biological performance interrelation of particular therapeutic MONPs. The development of functional, biocompatible and safe MONPs used in nanomedicine treatment of human diseases should be based on the proper understanding of all interactions between regulatory and activating factors and toxicity mechanisms. If the molecular regulation mechanisms of redox- and other signaling pathways that are triggered by NPs are profoundly understood then improved control over the cell cycle, migration, proliferation, differentiation, angiogenesis and so forth, could be exercised.

Besides all disadvantages, a new avenue for chemotherapy, immunotherapy, bone and wound healing and so forth has been provided by the development of NPs. The continuous advances in the development of engineered MONPs supplement multimodal approaches based on new and sophisticated methods for therapy and diagnosis of pathologies. Devices of metal oxide NPs and nanotechnologies have proven to be beneficial not only in smart drug delivery but also as theranostic platforms of non-invasive imaging. Since current diagnostic technologies face difficulties in the fading of fluorescent dyes due to bleeding, fluorescent NPs can overcome these shortcomings [288]. Metal oxide nanomaterials make it feasible to achieve smart drug delivery systems for lethal diseases that demand site-specific treatment with minimal side effects. Researches in cancer therapy are vigorously seeking multi-therapy options and remote control of the functions of NPs. Since molecular profiling of tumors and other diseases is unique, the selection of targeting agents or treatment based on the molecular profile will pave an avenue for targeted personalized medicine with a highly appropriate therapeutic strategy. This will open new doors to providing safer, more effective and customizable treatment options soon. This new area of individualized medicine will allow speeding up clinical trials and health promotion of cancer patients.

## Figures and Tables

**Table 1:** Type, size, characteristics of the biosynthesis and activity of the MONPs against different tested microorganisms.

MO	Precursor	Biosynthesis	NP Size [nm]	Tested Organism	Biological Activity/ Effect/Outcome	Ref.
**Biosynthesis of MONPs from plants**						
CuO	Cu_S_O_4_	Leaf extract of *Eichhornia crassipes*	20–22	*S. pneumonia—S. aureus*, *K. pneumonia*	- The highest inhibition zone was observed for *K. pneumonia*, the lowest for *S. aureus,* and moderate for *S. pneumonia*; - The bactericidal activity was almost equivalent to that of tetracycline.	[171]
CuO	Cu(O_2_CCH_3_)_2_	Stems of *Seidlitzia rosmarinus* ashes	8–40	*S. aureus*, *E. coli*	- Durable antibacterial activities against both *S. aureus* and *E. coli* on nano colored wool fabrics.	[172]
CuO	Cu(NO_3_)_2_. 3H_2_O	Aqueous leaf extract of *Abutilon indicum*	16.8	G− (*E. coli*) and G+ (*B. subtilis*, *S. aureus and Klebsiella*) bacteria	- At 5 mg concentration CuO NPs indicated effective antimicrobial activity against gram-positive bacteria; - In this concentration, CuO NPs were much more effective against *Klebsiella* and *B. subtilis* than standard drug ampicillin.	[162]
Cu_2_O	CuSO_4_.5H_2_O	Aqueous leaf extract of *Callistemon viminalis*	423	*E. coli*, *Acinetobacter baumannii*	- At a concentration of 512 and 1024 μg, *E. coli* strain was more susceptible to Cu_2_O NPs than *Acinetobacter baumannii*. - The zones of inhibition of cotrimoxazole and meropenem were larger for both bacteria than that induced by Cu_2_O NPs.	[173]
α-Fe_2_O_3_ γ-Fe_2_O_3_	Fe(NO_3_)_3_.9H_2_O	Leaf extract of *Platanus orientalis*	38	*Aspergillus niger*, *Mucor piriformis*	- Significant antifungal activity against *A. niger* and *M. piriformis* but more active against *M. piriformis.*	[174]
Fe_3_O_4_	FeCl_3_	Seeds, leaves and fruits of *Lagenaria siceraria*	30–100	*E.coli*, *S. aureus*	- Moderate antimicrobial activity when compared to the reference drug; - Higher bactericidal activity against *E. coli* and moderate against *S. aureus.*	[175]
Fe_3_O_4_	FeSO_4_	Flower sheath extract of *Musa ornate*	43.69	*S. aureus*, *Streptococcus agalactiae*, *E.coli*, *Salmonella enterica*	- Definite antibacterial activity against all tested bacteria without significant differences except in the case of *S. enterica* where the bactericidal effect was lower in all 5, 10, 15 and 20 mg mL^−1^ concentrations.	[176]
ZnO	Zn(O_2_CCH_3_)_2_	Plant extract of *Passiflora caerulea*	30–50	*Klebsiella* sp., *E.coli*, *Enterococcus* sp., *Streptococcus* sp.	- Maximum zone of inhibition against gram-negative *E. coli* and minimum zone against gram-positive *Enterococcus* sp.	[177]
ZnO	Zn(NO_3_)_2_	Aqueous leaf extract of *Solanum nigrum*	20–30	*S. aureus*, *Salmonella paratyphi*, *Vibrio cholerae*, *E. coli*	- The higher antimicrobial activity was found against *S. paratyphi* compared to the standard tablet; - less bactericidal activity was found for *S. aureus, V. cholera* and *E. coli* than a standard tablet. - The leaf extract also showed antimicrobial activity against *V. cholera and S. aureus*.	[178]
ZnO	Zn(O_2_CCH_3_)_2_	Leaf powder aqueous extract of *Scadoxus multiflorus*	31 ± 2	*Aedes aegypti* (larvae and eggs); *Aspergillus niger*; *Aspergillus flavus*	- Less larvicidal activity when compared to the literature; - 96.4% at 120 ppm ovicidal activity of the mosquitos; - small fungicidal activity against *A. niger* and *A. flavus.*	[179]
ZnO	Zn(O_2_CCH_3_)_2._ (H_2_O)_2_	Leaf extract *Atalantia monophylla*	20–45	Bacterial (*B. subtilis*, *B. cereus*, *S. aureus*, *P. aeruginosa*, *Klebsiella pneumonia*) and fungal species (*C. albicans*, *A. niger*)	- The biosynthesized ZnO NPs were found to outsmart conventional antibiotics and plant extracts in the destruction of pathogenic microorganisms. - Bacterial strains had a high susceptibility to ZnO NPs when compared to fungi.	[180]
ZnO	Zn(O_2_CCH_3_)_2._ (H_2_O)_2_	Green tea leaves (*Camellia sinensis*)	30–40	G+ (*S. aureus*) and G− (*E. coli*) bacteria; fungal species (*A. niger*)	- The better antibacterial activity of ZnO NPs (100 mg mL^−1^) than standard antibiotic (gentamycin—100 mg mL^−1^); - Better biocidal activity than other researchers findings and low MIC.	[181]
ZnO	Zn(O_2_CCH_3_)_2._ (H_2_O)_2_	Aqueous extract of parsley *(Petroselinum crispum)*	50 nm (at RT) 40 nm (at 90 °C)	*E. coli*	- The zone of inhibition was 4.8 mm at 90 °C and 4.3 mm at room temperature for ZnO NPs.	[182]
ZnO	ZnSO_4_	Leaf extract of *Bauhinia tomentosa*	22–94	G− (*P. aeruginosa*, *E. coli*) and G+ (*B. subtilis*, *S. aureus*)	- The higher bactericidal effect against gram-negative than gram-positive.	[183]
ZnO	Zn(O_2_CCH_3_)_2_	Leaf extract from *Stevia*	10–90	Parasitic strain: *Leishmaniasis major* Bacteria: *S. aureus* and *Escherichia coli*	- Low concentrations of ZnO NPs were required for the complete prevention of growth of these organisms in vitro.	[184]
ZnO and Cu- doped ZnO	Zn(NO_3_)_2_.6H_2_O and Cu(NO_3_)_2_	- Aqueous leaf extract of *Abutilon indicum* (for ZnO NPs) Extracts of *Clerodendrum infortunatum* (1 for Cu-doped ZnO NPs) *Clerodendrum inerme* (2 for Cu-doped ZnO NPs);	ZnO—16.7; Cu-doped ZnO method 1–17.5; Cu-doped ZnO method 2–20.7	Bacteria: *S. aureus*, *B. subtilis*, *Klebsiella*, *E. coli*; fungal strains *A. niger*, *A. flavus*, *Trichoderma harzianum*; An anticancer activity using human breast carcinoma cells	- ZnO NPs and Cu-dopped ZnO (method 1) showed effective antibacterial activity against *Klebsiella* and *B. subtilis*; - Cu-dopped ZnO (method 2) indicated superior and very effective broad-spectrum antibacterial activities because of higher Cu-doping and larger surface area; - Antifungal potential against *A. niger* and *T. harzanium* was observed more in method 2 MONPs than in method 1; - Cu-doped ZnO from method 1 and 2 showed a higher death rate for cancer, in contrast to control (standard drug).	[185]
RuO_2_	RuCl_3_.xH_2_O	Plant extract of *Acalypha indica*	6–25	*E. coli*, *P. aeruginosa*, *Serratia marcescens* *S. aureus*	- High antibacterial activity against both gram-negative (*E. coli*, *P. aeruginosa* and *Serratia marcescens*) and gram-positive (*S. aureus*) bacteria.	[186]
CeO_2_	CeCl_3_	Leaf extract of *Gloriosa superba L*.	5	*E. coli*, *S. aureus*, *S. dysenteriae*, *P. aeruginosa*, *P. vulgaris*, *K. pneumonia*, *S. pneumoniae*	- At 100 mg CeO_2_ NPs showed the most significant effect on the zone inhibition of *S. aureus. P. aeruginosa*, *P. vulgaris*, *K. pneumonia*, *S. pneumonia* and *E. coli* showed similar inhibition zone with 50 mg NPs; - Gram-positive bacteria are relatively more susceptible to NPs than gram-negative.	[187]
NiO	NiO(CH_3_COO)_2_.4H_2_O	Citrus fruit juice of *Limoinia acidissima Chrism*	20	G+ (*S. aureus*) and G− (*P. aeruginosa*, *E. coli*, *K. pneumonia*) bacteria	- The highest was the bactericidal activity against *E. coli* followed by *S. aureus*; - Less antibacterial activity was recorded against *K. pneumonia* and *P. aeruginosa.*	[188]
**Biosynthesis of MONPs from bacteria, fungi, algae and natural compounds**						
ZnO	ZnO powder	Culture of bacteria *Aeromonas hydrophila*	42–64	*Aeromonas hydrophila*, *E. coli*, *S. aureus*, *P. aeruginosa*, *Enterococcus faecalis*, *Streptococcus pyogenes*; *Aspergillus flavus*, *A. niger*, *C. albicans*	- The maximum zone of inhibition was observed in ZnO NPs against *P. aeroninosa* and *A. flavus*; - *A. hydrophila, E. coli, E. feacalis* and *C. albicans* showed minimum inhibition concentration at 1.2, 1.2, 1.4 and 0.9 μg mL^−1^ for the MONPs.	[189]
ZnO	Zn(NO_3_)_2_	Culture of *Bacillus megaterium*	45–150	*Helicobacter pylori*	- ZnO NPs exhibit high biocompatibility against hMSC and proved to be potentially safe in mammalian cells; - Anti-*H. pylori* dosage of ZnO NPs was safe to human mesenchymal stem cells (hMSC) and could effectively be used as a nano-antibiotic.	[190]
TiO_2_	TiO(OH)_2_	Mycelium of *Aspergillus flavus*	62–74	*S. aureus*, *E. coli*, *P. aeruginosa*, *Klebsiella pneumoniae*, *B. subtilis*	- The MIC of TiO_2_ NPs was 40 μg mL^−1^ for *S. aureus*, 40 μg mL^−1^ for *E. coli*, 80 μg mL^−1^ for *P. aeruginosa*, 70 μg mL^−1^ for *K. pneumoniae* and 45 μg mL^−1^ for *B. subtilis*.	[191]
ZnO	ZnCl_2_	A fungal isolate of *Aspergillus niger*	41–75	*S. aureus*, *E. coli*	- The effects of ZnO NPs against gram-positive were higher than that against gram-negative bacteria; - The inhibitory effect increased with concentration; - The antibacterial activity of ZnO NPs was comparable to that of conventional antibiotic ciprofloxacin (0.5 mg mL^−1^).	[192]
ZnO	Zn(NO_3_)_2_	Culture medium of *Aspergillus niger*	84–91	*S. aureus*, *E. coli*	- Impregnated fabrics with ZnO NPs showed zones of inhibition around 12 and 10 mm against *S. aureus* and *E. coli*, respectively.	[193]
CuO and Cu_2_O	CuSO_4_	Algae extract of brown algae *Bufurcaria bufurcata*	5–45	*Enterobacter aerogenes*, *S. aureus*	- The radial diameters of the inhibition zone of *E. aerogenes* and *S. aureus* were 14 and 16, respectively, therefore, gram-negative seemed to be more resistant to CuO NPs than gram-positive bacteria; - Significant antibacterial activity against both gram classes bacteria.	[194]
CuO	Cu(O_2_CCH_3_)_2._ H_2_O	Algae extract of cyanobacteria *Spirulina Platensys*	30–40	G−*: E. coli*, *Proteus vulgaris*, *Klebsiella pneumonia* G+*: S. aureus*; *S. epidermidis*, *Bacillus cereus*	- The largest radial diameter of inhibition for gram-negative bacteria was in *P. vularis* (around 28 mm); - The maximum inhibitory effect against gram-positive was found in *B. cereus* (around 27 mm).	[195]
ZnO	Zn(NO_3_)_2._6H_2_O	Algal extract of marine microalgae *Sargassum multicum*	4–23	*S. aureus—*sensitive and resistant; *C. albicans*—sensitive and resistant;	- The inhibition zone in both sensitive and resistant strains was comparable; - The zones of inhibition were about 28 and 25 mm for *C. albicans* and *S. aureus*, respectively; - The bactericidal activity of cotton fabrics impregnated with ZnO NPs was reduced by 2–25% after washing.	[196]
ZnO	ZnNO_3_.6H_2_O	Al-gum extrudates of *Azadirachta indica*	30–60	*E. coli*, *S. aureus*	- All tested bacteria showed resistance to ZnO NPs synthesized by the green method as compared with bulk ZnO; - At concentration 10 μg mL^−1^, both gram-positive and gram-negative bacteria indicated good sensitivity.	[197]
CuO	CuSO_4_	Goat (GFM) and sheep (SFM) fecal matter	29.2 ± 15.9 for GFM; 32.3 ± 32.2 for SFM	*Salmonella typhi*, *B. subtilis*	- CuO (GFM and SFM) NPs demonstrated significant antimicrobial activity against both bacteria compared to ampicillin.	[198]

## Abbreviations

A549	Adenocarcinomic human alveolar basal epithelial cells
AB	Antibiotic
ADMA	Exogenous asymmetric dimethylarginine
Ag	Silver
APTES	3-aminopropyl triethoxy saline
ATP	Adenosine triphosphate
Au	Gold
BBB	Blood-brain barrier
BC	Bacterial cellulose
BSA	Bovine serum albumin
CMX	Corona of the fusion protein
CPR	Cardiopulmonary resuscitation
Cr	Chromium
DMF	Dimethylformamide
DNA	Deoxyribonucleic acid
FTL	Ferritin light chain (a biomarker for ageing)
G(-)	Gram-negative
G(+)	Gram-positive
HeLa	Henrietta Lacks (human cell line)
HFL1	Human fetal lung fibroblast-1
HIV	Human immunodeficiency virus
HMSCs	Human mesenchymal stem cells
IC50	Half-maximal inhibitory concentration
IL-1α	Interleukin 1 alpha (hematopoietin)
IL-4	Interleukin 4
ITO	Indium tin oxide
Ka	Association constant
Kmapp	Michaelis–Menten constant
L-132	Human lung epithelial cell
MCF-7	Michigan cancer foundation-7 (cancer cell line)
MO	Metal oxide
MONPs	Metal oxides nanoparticles
MRI	Magnetic resonance imaging
MTT	Methylthiazolyldiphenyl-tetrazolium bromide salt
nm	nanometer
NO	Nitrogen monoxide
NP	Nanoparticle
O2	Oxygen
PAMAM	Poly (amidoamine)
PMMA-AA	Polymethyl methacrylate–acrylic acid
PBS	Phosphate-buffered saline
PCL	Polycaprolactone
PEG	Polyethylene glycol
PEI	Polyethylenimine
pH	Power of hydrogen
ppm	Parts per million
QDs	Quantum dots
RNA	Ribonucleic acid
ROS	Reactive oxygen species
RT	Room temperature
SDS	Sodium dodecyl sulphate
-SH	Thiol groups
SH-SY5Y	Human neuroblast cells
siRNA	Small (or short) interfering RNA
SPAN	Self-doped polyaniline nanofibers
SPR	Surface plasmon resonance
T-cell	T-lymphocyte
THP-1	Human monocytes
TNF-α	Tumor necrosis factor-α (inflammatory cytokines)
U mL-1	Units per millilitre
UV	Ultra-violet
VLP	Virus-like particles
